# Traditional protocols and optimization methods lead to absent expression in a mycoplasma cell-free gene expression platform

**DOI:** 10.1093/synbio/ysac008

**Published:** 2022-05-21

**Authors:** Andrei Sakai, Christopher R Deich, Frank H T Nelissen, Aafke J Jonker, Daniela M de C Bittencourt, Christopher P Kempes, Kim S Wise, Hans A Heus, Wilhelm T S Huck, Katarzyna P Adamala, John I Glass

**Affiliations:** Department of Genetics, Cell Biology and Development, University of Minnesota, Minneapolis, MN, USA; Institute for Molecules and Materials, Radboud University, Nijmegen, The Netherlands; Institute for Molecules and Materials, Radboud University, Nijmegen, The Netherlands; Synthetic Biology & Bioenergy, J. Craig Venter Institute, La Jolla, CA, USA; Embrapa Genetic Resources and Biotechnology, National Institute of Science and Technology—Synthetic Biology, Brasília, DF, Brazil; Santa Fe Institute, Santa Fe, NM, USA; Synthetic Biology & Bioenergy, J. Craig Venter Institute, La Jolla, CA, USA; Institute for Molecules and Materials, Radboud University, Nijmegen, The Netherlands; Institute for Molecules and Materials, Radboud University, Nijmegen, The Netherlands; Department of Genetics, Cell Biology and Development, University of Minnesota, Minneapolis, MN, USA; Synthetic Biology & Bioenergy, J. Craig Venter Institute, La Jolla, CA, USA

**Keywords:** mycoplasma, cell-free expression system, ribonuclease, *in vitro* transcription, *in vitro* translation

## Abstract

Cell-free expression (CFE) systems are one of the main platforms for building synthetic cells. A major drawback is the orthogonality of cell-free systems across species. To generate a CFE system compatible with recently established minimal cell constructs, we attempted to optimize a *Mycoplasma* bacterium-based CFE system using lysates of the genome-minimized cell JCVI-syn3A (Syn3A) and its close phylogenetic relative *Mycoplasma capricolum* (Mcap). To produce mycoplasma-derived crude lysates, we systematically tested methods commonly used for bacteria, based on the S30 protocol of *Escherichia coli*. Unexpectedly, after numerous attempts to optimize lysate production methods or composition of feeding buffer, none of the Mcap or Syn3A lysates supported cell-free gene expression. Only modest levels of *in vitro* transcription of RNA aptamers were observed. While our experimental systems were intended to perform transcription and translation, our assays focused on RNA. Further investigations identified persistently high ribonuclease (RNase) activity in all lysates, despite removal of recognizable nucleases from the respective genomes and attempts to inhibit nuclease activities in assorted CFE preparations. An alternative method using digitonin to permeabilize the mycoplasma cell membrane produced a lysate with diminished RNase activity yet still was unable to support cell-free gene expression. We found that intact mycoplasma cells poisoned *E. coli* cell-free extracts by degrading ribosomal RNAs, indicating that the mycoplasma cells, even the minimal cell, have a surface-associated RNase activity. However, it is not clear which gene encodes the RNase. This work summarizes attempts to produce mycoplasma-based CFE and serves as a cautionary tale for researchers entering this field.

Graphical Abstract

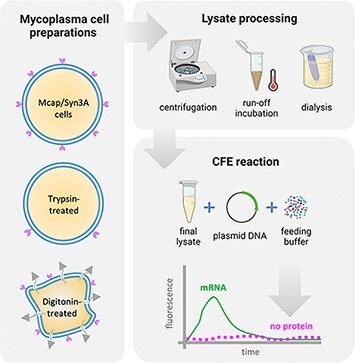

## 1. Introduction

One of the greatest challenges in modern science is to bring inanimate matter to life. Achieving such a transition would shed light on the very principles by which complex sets of chemical reactions create living systems. This key milestone will create new directions for research into synthetic systems that could lead to completely novel, yet unexplored, complex systems that have the ability to evolve. There is a general consensus that the minimal living system needs to be a cell. A key stumbling block in the quest to create a minimal living cell from the bottom up is the sheer complexity of even the simplest living systems and our lack of knowledge of the general design principles that would allow us to construct a roadmap toward the bottom-up construction of a minimal cell. Most efforts to the bottom-up construction of a synthetic cell are based on *Escherichia coli* cell-free expression (CFE) systems, using either the cell lysate or the Protein synthesis Using Recombinant Elements (PURE) system (1, 2). While *E. coli* is a valuable model for many purposes, the organism is vastly complex.

In contrast, more simplified cellular systems are now available through the application of top-down synthetic biology strategies to reduce genome content (3–5). One approach is to redesign and rebuild the genomic software of a cell *ex vivo* and reintroduce it into a compatible recipient host through genome transplantation (6), thereby redirecting the cellular machinery to create a new cell with reprogrammed attributes. The J. Craig Venter Institute (JCVI) pioneered this strategy and applied it to completely synthesize, then minimize, a genome based on a template sequence of a natural organism of the genus *Mycoplasma* (hereafter mycoplasmas): *Mycoplasma mycoides* subspecies *capri* (7). The initial cell construct JCVI-syn1.0 (hereafter Syn1.0) has a genome of 1079 kb, similar to that of its natural precursor. Extensive genome reduction of Syn1.0 resulted in the ‘minimal bacterial cell’ JCVI-syn3.0 (hereafter Syn3.0), with a genome of 531 kb comprising 473 genes (8). This is a vastly simpler organism than *E. coli* K-12, whose >4600-kb genome contains >4200 genes (9). While Syn3.0 propagates in the laboratory, its slow growth rate and pleomorphic phenotype render it unsuitable for some applications. To overcome this, a near-minimal cell JCVI-syn3A (hereafter Syn3A) restoring 19 genes that are not present in Syn3.0 has been constructed as a more robust laboratory model and is in use to study various aspects of cell biology (5, 10, 11). The additional genes include a subset required for proper cell division, others of completely unknown function and a ribosomal RNA (rRNA) operon that restores its redundant presence in the original Syn1.0 genome (10).

Our strategy to construct a bacterial cell from nonliving parts leverages the lessons learned from the genome transplantation process used to boot up isolated *M. mycoides* and minimized *M. mycoides* genomes. In genome transplantation, a donor genome that is constructed as a yeast centromeric plasmid is installed in a recipient cell in a way that it commandeers that cell to produce bacteria programmed only by the donor genome (6, 12). To date, the only acceptable genome transplantation recipient cell is *Mycoplasma capricolum* subspecies *capricolum* (hereafter Mcap). The only donor genomes that work are all close phylogenetic relatives of Mcap (13). This is presumably necessary because the software of the donor genome has to be capable of properly interacting with the enzymatic machinery in the recipient cell cytoplasm, such as the ribosomes or polymerases. By using donor genomes isolated from yeast instead of *M. mycoides* bacteria, there is no possibility of a resulting transplant being a contaminating cell that somehow made it through the chromosome isolation process. By transplanting genomes from one species of mycoplasma into a recipient cell from a different species, sequence analysis of any genome transplant can confirm whether the transplant cell genome is the result of recombination between the donor genome and the recipient cell genome. Our plan to construct a synthetic cell from nonliving parts is to fill micron-sized lipid vesicles with cytoplasm derived from bacteria and then install a genome in that CFE containing vesicle in a way resulting in the enlivening of the assembled parts. Given the similarity of this plan to the genome transplantation technique, our intent is to use the same reagents, controls and safeguards that are part of the genome transplantation protocol. We plan to use a Syn3A genome obtained from a yeast as the genetic software of our synthetic cell and a CFE derived from Mcap.

Here, we report our attempts to develop a CFE system using these cells, starting with protocols developed for successful *E. coli* lysates. We will discuss how unexpected nuclease activity in the lysate initially prohibited transcription, even to the extent that Mcap lysate poisoned *E. coli* CFE. We overcame these problems, but despite our best efforts and testing every known procedure for lysate preparation, there appear to be as yet unknown aspects of mycoplasma biology that prevent us from obtaining a functional lysate.

Considering the intense interest in the bottom-up construction of synthetic cells in general, our work should serve as a general reminder of the many complexities that will be encountered in these endeavors. We are aware that our results do not bring a minimal genome-based synthetic cell closer. However, we identify several factors that limited our success that may inform such efforts in other organisms. We also discuss alternative approaches for packaging the cell machinery of minimal cells to create an operational bottom-up synthetic cell.

## 2. Materials and methods

### 2.1 Reagents

Chemicals and reagents were purchased from Sigma Aldrich (reagent-grade), except those specified later.

### 2.2 Microorganisms and cell culture

For the preparation of mycoplasma cell-free extracts, we cultivated the mycoplasma strains Mcap and Syn3A (6, 8). Among mycoplasma species, Mcap presents a faster and more robust growth than Syn3A. This difference in growth rates is explained by the significant genome reduction in Syn3A (543 kb) compared to its parent genome (1079 Mb, JCVI-syn1.0). As obligate parasites, mycoplasmas require a rich medium (including serum) for *in vitro* growth. Mycoplasma cells were cultured in adapted SP4 glucose broth including 17 vol% KnockOut™ serum (Gibco) as a replacement for fetal bovine serum (SP4-KO medium) (14, 15). For lysates prepared by digitonin treatment, cells were grown in a modified Hayflick medium supplemented with 20 vol% heat-inactivated horse serum (ThermoFisher) (HS medium) (16). Cells were incubated at 37°C without agitation or aeration. A fully grown culture of 500 ml volume required about 6 days from the inoculation of a 1-ml culture (in a 14-ml tube), subsequent transfer into a 100-ml culture (in 250-ml flasks) and the final transfer into a 500-ml culture (in 2-l flasks). Cell growth was determined by color change (phenol red) as proliferating cells accumulate acidic metabolic by-products. *E. coli* lysates were prepared from the BL21 (DE3) Star™ pLysS strain containing plasmid to express rare transfer RNAs (tRNAs) (pRARE, Invitrogen) following a protocol previously described (17).

### 2.3 Lysate preparation

**Figure 1.**
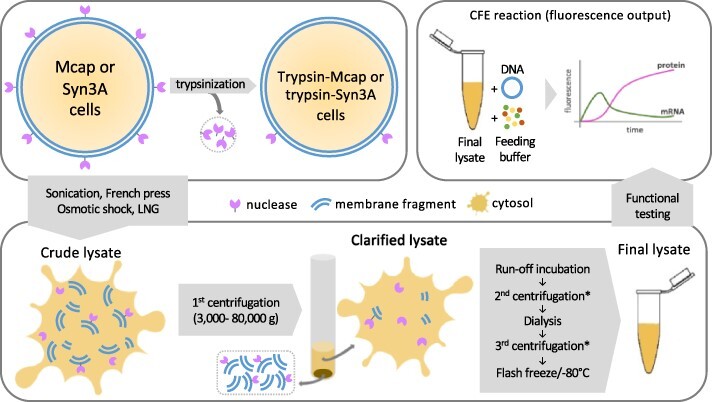
Summary of conditions tested for the preparation of mycoplasma lysate and its functional assessment. The lysate preparation starts with harvesting mycoplasma cells (upper left). For some preparations, cells were trypsinized before cell disruption. To obtain crude lysates, we initially tested different cell lysis methods: sonication, French press, osmotic shock and liquid nitrogen grinding (lower left). In the first centrifugation step, most of the cell debris are decanted, and the supernatant (clarified lysate) proceeds to run-off incubation, second centrifugation, dialysis (in S30B buffer) and third centrifugation. The final lysate is then flash-frozen and stored at −80°C (lower right). To test lysate functionality, we performed CFE reactions and measured transcription and translation by tracking fluorescent probes. Some lysate preparations included cell trypsinization before lysis. *The second and third centrifugation speeds are the same as the first centrifugation step.

*E. coli* CFE is currently the CFE system with the best protein production levels (18). The successful preparation of *E. coli* crude lysates for the CFE system, known as the S30 protocol, started many decades ago (19–22), and it has been gradually improved (23, 24). Each *E. coli* lysate batch has a slightly different composition and consequently a distinct efficiency regarding the protein production. In this work, we used the same batch of *E. coli* extract within a single experiment but different batches between experiments (due to extract availability). Since there is no reported protocol for the preparation of mycoplasma lysates for the CFE system, we adapted a recent S30 protocol (17) for the preparation of mycoplasma lysates. Mcap or Syn3A were harvested at the stationary growth phase (culture medium pH ∼5) by centrifugation at 5000 g, 4°C for 15 min. Pellets were washed twice with the cold S30A buffer (50 mM Tris pH 7.7, 14 mM Mg-glutamate, 60 mM K-glutamate), keeping the flasks as much as possible on ice (ideally also in the cold room). The original S30 protocol consists of (i) cell lysis (by French press or sonicator, Figure 1) followed by (ii) run-off incubation to release ribosomes from polysomes and (iii) dialysis for buffer exchange (S30B buffer: 5 mM Tris pH 8.2, 14 mM Mg-glutamate, 60 mM K-glutamate). High-speed centrifugation steps are employed to clarify the lysate after the lysis, the run-off incubation and the dialysis steps (at the same speed). Since the original protocol generates clean supernatants at a 30 000-g centrifugal force, this method is known as S30.

Because of morphological differences between *E. coli* and mycoplasmas (e.g. size and cell membrane composition), we tested different methods for cell lysis: (i) sonication at 150–1000 J/ml (Q500, 40% amplitude, 3 mm tip, Fisherbrand); (ii) French press at 140–360 mPa (FPG12800, Homogenizing Systems Ltd); (iii) liquid nitrogen grinding with mortar and pestle, 20 min non-stop with the frequent addition of liquid nitrogen; (iv) osmotic shock by incubation in S30B buffer, supplemented with 250 mM NaCl (30 min, 23°C) or (v) by treatment with detergents, see Section 2.6. The lysate is typically centrifuged at 20 000 g for 20 min at 4°C to remove cellular debris (a first clarification step). Next, the supernatant is incubated at 37°C for 15–80 min (run-off incubation), followed by a second clarification step (20 000 g, 4°C, 20 min). The supernatants were then dialyzed in 10 kDa molecular weight cutoff (MWCO) cassettes (Slide-A-Lyzer™ ThermoFisher) against S30B buffer (0.5× final concentration) (17). Samples were clarified once more by centrifugation (20,000 g, 4°C, 20 min) to remove cellular debris. After centrifugation the supernatant was collected and flash frozen in liquid nitrogen before final storage at −80°C.

### 2.4 Mycoplasma cell surface trypsinization

Mycoplasma cell cultures were centrifuged at 5000 g for 10 min at 4°C. The supernatant was discarded, and the pellet was washed with 75 mM HEPES pH 8.0 buffer. The washed pellet was again centrifuged (5000 g, 10 min, 4°C), and the supernatant was discarded. The washed pellet was then resuspended in 75 mM HEPES pH 8.0 buffer supplemented with 100 µg trypsin (Merck Life Science) per 100 mg of cell pellet and incubated at 37°C for 30 min under mild agitation (100 rpm). Afterward, trypsinized cells were centrifuged at 5000 g for 10 min at 4°C, and the supernatant was discarded. The final pellet was washed once with purified soybean trypsin inhibitor solution (Gibco, 1× defined trypsin inhibitor, #R007100), centrifuged at 5000 g for 10 min at 4°C and finally washed with 75 mM HEPES pH 8.0 buffer. Alternatively, trypsin was inhibited using the SP4-KO medium.

### 2.5 Separation of cells and media from mycoplasma cultures by sucrose cushion

Mycoplasma cell suspensions or cell culture media (400 µl, Mcap or Syn3A) were underlaid with a sucrose cushion (600 µl, 0.5 M sucrose in 20 mM HEPES pH 8, filter-sterilized) and centrifuged at 3500 g for 10 min. The supernatant (culture medium or cell suspension buffer) was carefully removed by vacuum aspiration using a Pasteur pipette. The cell pellet was carefully resuspended and used for subsequent experiments.

### 2.6 Lysate preparation by Triton™ X-100 or digitonin treatment

Mcap or Syn3A were cultured in the HS medium (2-l flasks), as described in Section 2.2, and harvested at pH 5.5–6 by centrifugation at 5000 g, 4°C for 15 min. Next, cell pellets were washed twice with the fresh HS medium and collected by centrifugation (7000 g, 4°C, 15 min). Cells were lysed upon resuspension with a lysis buffer (1 ml/100 mg of cell pellet) containing 1 vol% of Triton™ X-100 (hereafter TX100) and incubation on ice for 15 min (with vortexing every 5 min). The lysis buffer also included 75 mM HEPES pH 8.0, 1× HALT™ protease inhibitor cocktail without ethylenediaminetetraacetic acid (EDTA) (ThermoFisher), 20 mM CaCl_2_, 1 mM polyvinyl sulfonic acid MW ∼2–5 kDa (PVSA) (25). The crude lysate was clarified by high-speed centrifugation (32 000 g, 4°C, 20 min). When recovering the supernatant, we avoided to collect the pellet or any floating debris. Afterward, the clarified lysate was concentrated 10-fold with a centrifugal filter unit (Merck Millipore Amicon™ 15 ml, 10 kDa MWCO), followed by dialysis (Spectra™ 3.5 kDa MWCO dialysis membrane) against 0.5× S30B buffer at 4°C for 2 h. Run-off incubation was not performed. Finally, samples were flash-frozen and stored at −80°C.

For the preparation of mycoplasma lysates using digitonin, we used a similar protocol described for TX100 except for the surfactant in the lysis buffer (1 mg/ml of digitonin instead of 1 vol% of TX100). To improve the solubility of digitonin in an aqueous solution, we prepared a stock of 2 wt% digitonin in deionized water and heated it to 95°C for 5 min. The solution was allowed to cool down to room temperature before mixing with other components of the lysis buffer.

### 2.7 Total RNA analysis

RNA was isolated from mycoplasma cells or lysate by the hot phenol extraction method (26). Isolated RNA fractions were resuspended in nuclease-free water, and their concentration was measured by spectrophotometry (Nanodrop™, ThermoFisher). RNA was diluted to a concentration of 1 µg/µl, and 5 µl of each sample was analyzed by performing denaturing polyacrylamide gel electrophoresis (8% acrylamide/bis-acrylamide, 8 M urea, 0.5× Tris/Borate/EDTA buffer, 250 V for 45 min). Gels were stained with SYBR^TM^-Gold (Invitrogen). Alternatively, total RNA fractions were analyzed using chip-based capillary electrophoresis in agarose gel (RNA 6000 Nano kit, Agilent 2100 Bioanalyzer). For that, samples were diluted to a 500-ng/µl final concentration, of which 1 µl of each sample was analyzed in the chip. The Bioanalyzer total RNA plots show arbitrary fluorescence units (*y*-axis) versus migration time (*x*-axis). For better data interpretation, the *x*-axis was converted to RNA size using an RNA ladder as reference (25–6000 nt). RNA was converted into a cDNA library (iScript^TM^, Bio-Rad) for the quantitative polymerase chain reaction (qPCR) experiment (iQ^TM^ SYBR^®^ Green Supermix, Bio-Rad). qPCR was performed with primers for 23S rRNA (see Supplementary Table S4 for sequences).

### 2.8 Cell-free expression

Reactions mixtures were designed from the *E. coli* CFE system previously described (17). Bacterial lysates can show batch-to-batch variation in CFE efficiency. Therefore, for each CFE experiment, we used a single lysate batch to generate consistent results. The reaction mix comprised cell lysates (33 vol%), feeding buffer (18.5 vol%) and a suspension of DNA template (plasmid or linear DNA, 6–10 nM, 5–10 vol%) adjusting the final volume with deionized water or S30B buffer. GamS nuclease inhibitor (3 µM final concentration) was added when linear DNA was used as a template (27). The feeding buffer (Supplementary Table S1) contains the amino acid mixture (1.5 mM of all 20 amino acids initially dissolved in 5 M KOH and finally adjusted to pH 6.52 with acetic acid) and energy solution (50mM HEPES pH 8, 1.5 mM ATP and GTP, 0.9 mM CTP and UTP, 0.2 mg/ml, *E. coli* or *Saccharomyces cerevisiae* tRNA, 0.26 mM CoA, 0.33 mM NAD, 0.75 mM cAMP, 0.07 mM folinic acid, 1 mM spermidine, 30 mM phosphoglyceric acid (3-PGA), 1 mM DL-dithiothreitol (DTT)).

Mg-glutamate (5–30 mM), K-glutamate (60–120 mM), RNase inhibitor (60 U/150 µl reaction mix volume of recombinant RNase inhibitor, ThermoFisher #N8080119) and recombinant T7 RNA polymerase (120 U/150 µl of reaction mix volume) were added to all reactions. For some reactions, polyethylene glycol 8000 (2 vol%, PEG8000, MW ∼8000 kDa) and calcium salts (calcium chloride, calcium acetate, calcium glutamate, 0–25 mM) were added. Two alternative RNase inhibitors, Superase-In and RNase Off, were also used at 60 U/150 µl. Samples were analyzed in a microplate reader (M200 or M10 Tecan, SpectraMax Gemini, fluorescence detection) using black 384-well plates with a flat and transparent bottom (Greiner Bio-One, #781900, 11 µl samples per well). Green fluorescent protein (GFP) was analyzed at 488/525 nm (excitation/emission) and red fluorescent protein (mCherry) at 560/610 nm. The plate was kept at 30°C for 4–24 h (5 min data acquisition interval) at 30°C. Graphs were prepared using Origin 8 software.

### 2.9 Mycoplasma DNA constructs

Translation was monitored by the expression of mCherry or enhanced-GFP (eGFP) (Supplementary Figure S1). DNA template coding for mCherry under the control of the *Pspi* promoter was previously developed for mycoplasma *in vivo* experiments and provided by Prof. Yo Suzuki (28). The eGFP gene was optimized from the original *E. coli* sequence for mycoplasma usage (Supplementary Table S5) using an online codon usage database and an automated DNA sequence optimization tool (29, 30). Cloning was performed by Golden Gate assembly using a pRSET5d vector and transformed into XL-1 *E. coli* chemically competent cells (Agilent Technologies). Primers are listed in Supplementary Table S4.

For tracking transcription, we used malachite green (MG, Addgene, pJBL7004) and Spinach2 RNA aptamers combined with their respective dyes (Supplementary Table S5) (31–35). Spinach2 RNA aptamer utilized (5Z)-5-[(3,5-difluoro-4-hydroxyphenyl) methylene]-3,5-dihydro-2-methyl-3-(2,2,2-trifluoroethyl)-4H-imidazol-4-one dye (DFHBI-1T) and MG aptamer complexed with MG dye. For tracking RNA degradation, we used Broccoli RNA aptamer with 3,5-difluoro-4-hydroxybenzylidene imidazolinone dye (DFHBI) (33, 36). The reaction mix contained a 1–8 nM RNA aptamer template and a 60 µM DFHBI-1T.

### 2.10 mRNA degradation assay

The messenger RNA (mRNA) substrate (coding for eGFP, ∼800 nucleotides long, 1.5 µg) was mixed with mycoplasma or *E. coli* lysate (protein concentration ∼3 mg/ml) in a 5 µl final volume sample (volume adjusted with deionized nuclease-free water) kept in ice. Next, the samples were incubated at 30°C for 1–10 min to assay mRNA degradation. To quench RNAse activity after incubation, samples were diluted with TES buffer (10 mM HEPES pH 7.5, 10 mM EDTA, 0.5 vol% SDS) to a final volume of 100 µL. The RNA fraction was purified using the hot phenol extraction method (26). Dried RNA pellets were resuspended in 5 µl deionized water and mixed with 11 µl of sample buffer (0.85 mM EDTA, 12.5 mM 4-morpholinepropanesulfonic acid (MOPS), 3.1 sodium acetate, 25 vol% formamide, 63 vol% formaldehyde solution (37 wt% in water) and 0.25 µl ethidium bromide). RNA samples were analyzed using denaturing 1% agarose gels (60 V for 100 min in 1× MOPS as running buffer: 20 mM MOPS, 5 mM sodium acetate, 1 mM EDTA, pH 7.0).

The mRNA substrate was synthesized by *in vitro* transcription (IVT) in a reaction containing 40 mM Tris-HCl pH 8.1, 25 mM MgCl_2_, 5 mM DTT, 1mM spermidine, 4 mM ribonucleotide triphosphates (rNTPs), 5 mM guanosine-5′-monophosphate, 5 nM of a linear double-stranded DNA (dsDNA) template encoding the green fluorescent protein gene behind a T7 RNA polymerase promoter (T7p-GFP), and T7 RNA polymerase 600 U/500 µl reaction mix volume. The IVT reaction mix was incubated at 37°C for 4 h, followed by RNA precipitation with 97% ethanol and centrifugation for 15 min at 14 000 g. The mRNA pellet was washed once with 80% ethanol and then spin-dried to remove solvent. The dried pellet was resuspended in nuclease-free water (∼800 ng/µl).

### 2.11 Statistical analysis

Experiments were done in triplicate (*n* = 3) whenever feasible. Error bars in the figures are the standard deviation of multiple experiments.

## 3. Results

### 3.1 Development of a mycoplasma CFE platform

CFE platforms have been derived from various prokaryotes and eukaryotes (18). However, no protocols for the preparation of a mycoplasma lysate for a CFE platform have been reported in the literature. For the development of a robust CFE platform based on Mcap and Syn3A, we started out by systematically testing several conditions used in a well-established protocol for obtaining the robust CFE platform of *E. coli*, known as the S30 protocol (1, 17). It comprises cell lysis, extract clarification, run-off incubation and dialysis (Figure 1). The S30 protocol has been used by other researchers as a starting point to produce lysates from non-model prokaryotes, basically using the same essential steps. To date, no organism was able to yield similar protein production levels as the *E. coli* CFE platform. In terms of protein synthesis capacity, lysates derived from Gram-negative bacteria such as *Vibrio natriegens* and *Pseudomonas putida* have lower yields, by ∼35% and 90%, respectively, in batch reactions of GFP (37–39). When lysates were produced from Gram-positive bacteria, the protein yields were even lower. For instance, *Bacillus megaterium* and *Bacillus subtilis* remained <4% of *E. coli* CFE’s full protein synthesis capacity (18, 40, 41). Based on the 60-year history of the S30 protocol development and the phylogenetic differences between *E. coli* and mycoplasmas, we were aware of the complexity of creating the mycoplasma CFE platform from scratch.

We initially hypothesized that the mycoplasma lysate preparation would compare to the S30 lysate protocol with adjustments due to the unique cell membrane of mycoplasmas (i.e. absent cell wall and cholesterol-rich). We initially tested physical cell disruption methods (i.e. sonication, French press, liquid nitrogen grinding and osmotic shock), since these methods have been successful in producing active lysates for most prokaryote CFE platforms, including Gram-negative (*E. coli* and *V. natriegens*) and Gram-positive bacteria (*Bacilli*). Because of the absence of a cell wall in mycoplasmas, we also tested surfactant-based cell lysis methods (i.e. by TX100 or digitonin treatment). In addition to the lysis method, we also analyzed the importance of centrifugation speeds for lysate clarification, run-off incubation and dialysis on the lysate’s capacity to support transcription and translation (Table 1).

**Table 1.** Conditions tested for the cell lysis step for preparing mycoplasma lysates

Lysis method	Lysis condition	No. of cycles	Run-off incubation	Dialysis
Sonication	20%, 35%, 40%, 50% amplitude	1, 2, 4, 5, 7 (30 s on, 30/60 s off)	±	±
Osmotic shock	250 mM NaCl	2 washes, resuspend (30 min, 23°C)	+	+
Liquid nitrogen grinding	Grinding flash-frozen pellet	Liquid nitrogen added every minute (for 20 min)	+	–
French press	1000, 2000 psi	1, 3	±	±
Surfactant-based	1 vol% TX100 1 mg/ml digitonin	1–3 cycles (15 min, ice)	–	+

For CFE batch reactions, lysates were mixed with a feeding buffer and DNA template as described in Materials and Methods section 2.3 (Lysate preparation). Starting from the optimal concentrations for the *E. coli* CFE platform, we systematically tested a range of concentrations for key components in mycoplasma CFE (e.g. Mg-glutamate, K-glutamate, DNA template and PEG8000) (Table 2).

**Table 2.** CFE reaction mix compositions tested for the mycoplasma CFE platform, the standard concentration ranges for *E. coli* CFE are shown in the right column

Component	Mycoplasma CFE	Optimal *E. coli* CFE*
Mg-glutamate	0–25 mM	2.5–10 mM*
K-glutamate	60–120 mM	60–80 mM
DNA template	6–10 nM	5–8 nM*
Lysate buffer	1× S30B, 0.5× S30B, 75 mM HEPES pH 8.0	1× S30B, 0.5× S30B, deionized water
Lysate fraction	33–50 vol%	33 vol%*
Dilution solvent	1× S30B, 0.5× S30B, deionized water	Deionized water
RNase inhibitor	Recombinant RNase A/B/C inhibitors, RNAsecure®, PVSA	–

* The optimal concentrations of Mg-glutamate, DNA template and lysate fraction varied from batch to batch. Therefore, we display concentration ranges in which we observed the best protein yields.

For testing transcription, we used DNA template coding for the RNA aptamers MG and Spinach2 controlled by an exogenous T7 promoter. In presence of the respective dyes (MG dye complexed with MG aptamer and DFHBI-1T complexed with Spinach2 aptamer), we measured the fluorescence of RNA aptamer-dye complexes. For testing protein production, we prepared DNA template coding for eGFP and mCherry controlled by an exogenous T7 promoter (Supplementary Figure S1A) or an endogenous promoter (Supplementary Figure S1B). To ensure the maximum translation efficiency, eGFP and mCherry gene sequences were codon-optimized for mycoplasma usage (28–30).

We tested different cell disruption methods to find the best conditions for mycoplasma cell disruption. The functionality of each lysate was assessed in terms of its capacity to support transcription and translation. Cell disruption efficiency depends on the lipid composition of the cell membrane and the energy level employed for cell disruption (for mechanical disruption methods) (42, 43). Compared to other Gram-positive (e.g. *Bacilli*) and Gram-negative bacteria (e.g. *E. coli* and *Vibrio*), mycoplasma cells are relatively small (∼0.4 µm versus 2 µm *E. coli*), and their plasma membranes rely on cholesterol and cholesterol esters for mechanical stability rather than a cell wall (44, 45). Therefore, methods available to lyse *E. coli* or *Bacilli* may not be readily applicable to mycoplasma.

First, we tested a range of energy levels for mycoplasma cell disruption by sonication (150, 300, 500 and 1000 J). Sonicated Mcap lysates did not show CFE regardless of the energy level employed for lysis or centrifugal force (3000 and 12 000 g) (Supplementary Figure S2A–O). Lysis by osmotic shock (Supplementary Figure S2P) or liquid nitrogen grinding (Supplementary Figure S2Q) also failed to support CFE for Mcap as well as Syn3A. Different energy regeneration molecules (phosphoenolpyruvate (PEP) or 3-PGA) (Supplementary Figure S2A–L) or adding a molecular crowder (PEG8000) (Supplementary Figures S2A–O and S3) failed to improve the expression of DNA template coding for eGFP controlled by the T7 promoter in Mcap CFE. Similar results were observed using a DNA template coding for the mCherry sequence controlled by the *Pspi* promoter (Supplementary Figure S3) (28).

To understand the basis for our failure in setting up a working mycoplasma CFE system, we next investigated Mcap and Syn3A extracts for evidence of *in vitro* transcription only. The rationale for using Syn3A was that the minimized Syn3A genome contained only about half the number of genes present in Mcap, and we might be able to produce a minimal cell (i.e. Syn3A) extract being capable of CFE due to the absence of a nonessential nuclease or protease gene. Using linear dsDNA templates encoding a T7 RNA polymerase promoter, a low level of transcription was detected for the MG RNA aptamer expressed in *E. coli* lysate, but Mcap and Syn3A lysates prepared by liquid nitrogen grinding did not give a clear signal (Supplementary Figure S4), indicating no expression of MG aptamers in Syn3A or Mcap. These experiments suggested that poor transcription rates or rapid degradation of DNA-RNAs or a combination thereof prevented us from obtaining a functional CFE system using mycoplasma extracts.

To investigate the possible impact of RNA degradation on mycoplasma CFE, we measured the fluorescence signal of pre-transcribed Broccoli aptamer complexed with DFHBI dye in various extracts as a function of time (36). DFHBI-Broccoli rapidly degraded over time with a more pronounced decrease in Mcap lysate obtained by sonication than in Syn3A lysate obtained by sonication (Supplementary Figure S5B), irrespective of the amplitude used (Supplementary Figure S5A and B). In a kinetic assay, DFHBI-Broccoli degraded in Mcap lysate produced by sonication but not in Syn3A lysate produced by sonication (Supplementary Figure S5C). Degradation of DFHBI-Broccoli was even faster in the Mcap lysate produced by liquid nitrogen grinding (Supplementary Figure S5D). Nitrogen-ground Syn3A lysate degraded DFHBI-Broccoli over time but at a much slower rate. *E. coli* lysate controls made by sonication and liquid nitrogen grinding both degraded DFHBI-Broccoli. Different lysate preparation techniques might be responsible for the differences in DFHBI-Broccoli degradation that we see from Syn3A lysates made by sonication versus liquid nitrogen grinding. Liquid nitrogen grinding avoids overheating proteins during extraction, which could lead to denaturation and degradation. However, because the fluorescent signal from the aptamer-dye complex depends on the RNA folding, it cannot be ruled out that the low level of fluorescent signal is caused by improper folding in lysates as well. This could well explain the unexpected low signal of DFHBI-Broccoli in the *E. coli* lysate that was used as a positive control.

In summary, most of our mycoplasma extracts presented low levels of transcription and rapid degradation of reporter RNAs (except for Syn3A lysate, which degraded the DFHBI-Broccoli complex at a slower rate) were observed as well as for *E. coli* lysate. As a reference, the fluorescence signal of RNA-dye complex can be 2–10 times higher in *E. coli* CFE systems, depending on the type of RNA aptamer, dye, buffer composition and plate reader settings (46). The inability to develop a mycoplasma CFE system starting from successful protocols for *E. coli* lysates made us wonder if some yet unknown aspects of mycoplasma biology might pose additional challenges to the development of a mycoplasma CFE system. Therefore, we next explored the mycoplasma metabolism to identify factors that might affect the production of a functional CFE system.

### 3.2 Gene expression assay with *E. coli* lysate led to investigation of the critical impact of nuclease activity in mycoplasma CFE

Membrane-associated nucleases enable mycoplasmas to scavenge the extracellular environment for nucleotides. These membrane-associated nucleases might impair mycoplasma CFE by degrading essential nucleic acids involved in transcription and translation. Even though the enzymatic activity of membrane-associated nucleases for some mycoplasma species is described in the literature (47–52), little is known about their structure and inhibition mechanisms.

Mcap and Syn3A share 20 common nucleases with annotated functions (Supplementary Table S2). The only common nuclease for Mcap and Syn3A associated with the cell membrane is RNase Y, which is a degradosome protein C terminally anchored to the inner membrane related to RNA turnover mechanisms (53). It is possible that both Mcap and Syn3A contain unannotated surface nucleases. Many of the genes of unknown function in both organisms encode membrane-associated proteins.

**Figure 2.**
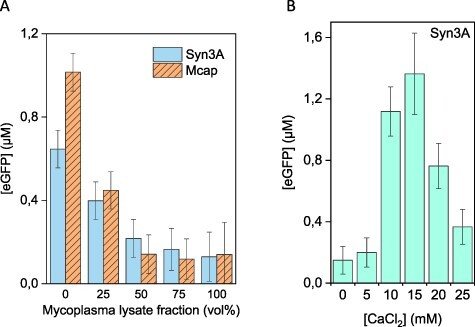
Effect of adding mycoplasma lysate and CaCl_2_ to *E. coli* CFE. (**A**) Expression of eGFP decreases by adding increasing amounts of Syn3A or Mcap lysate from 0% (100 vol% *E. coli* lysate) to 100% (100 vol% mycoplasma lysate). (**B**) Addition of CaCl_2_ to ∼15 mM restored eGFP expression in a 1:1 *E. coli*:Syn3A CFE reaction. An *E. coli* codon-optimized plasmid under the control of a T7 promoter was used for *E. coli* CFE reaction of eGFP.

To test mycoplasma lysates for factors incompatible with CFE, we added mycoplasma lysate directly to an *E. coli* CFE (Figure 2A). Since we aimed to measure the effect of mycoplasma lysate in normal *E. coli* CFE reaction, we employed a plasmid coding for eGFP controlled by T7 promoter codon-optimized for *E. coli*. Indeed, with increasing mycoplasma lysate fraction, eGFP expression by *E. coli* CFE was gradually decreased. Importantly, eGFP expression in *E. coli* CFE mixed with Syn3A lysate could be restored by adding the surface nuclease inhibitor CaCl_2_ (Figure 2B; SupplementaryTable S3). Even though CaCl_2_ is described as a specific inhibitor of Mcap nuclease activity (49) (what is true for Mcap is likely true for *M. mycoides*), we did not discard the possibility of other roles for CaCl_2_ in the CFE environment. In this instance, the effect of adding an extra component to the CFE reaction mix (i.e. mycoplasma lysate), likely did not have a significant effect on the GFP yields observed. As evidence, the GFP yields in a reaction containing 15 mM CaCl_2_ (50% Syn3A lysate in *E. coli* CFE reaction) were similar to the yields obtained in 100% *E. coli* CFE, which was around the maximum yield obtained for this reaction.

These results suggested a possible role of nucleases present in the mycoplasma lysates in ‘poisoning’ CFE and a possible remedy by adding CaCl_2_. A murine RNase inhibitor (NEB) was used throughout the experiments but did not increase the eGFP signal. Two other commercially available RNase inhibitors, Superase-In™ (ThermoFisher) and RNase Off™ (BioVision), were also examined for their capacity to inhibit RNA nuclease activity in mycoplasma lysates, but neither could produce measurable eGFP expression (Supplementary Figure S13A and B). Rescue of eGFP expression in *E. coli* CFE poisoned with mycoplasma lysate was unsuccessful using any of the RNases (SupplementaryFigure S13C).

Encouraged by the CaCl_2_ result, we tested expression in 100% Syn3A CFE reaction with CaCl_2_. However, no protein production was detected (Supplementary Figure S6A). Calcium acetate also failed to support eGFP synthesis in either a 100% Syn3A or 100% Mcap CFE reaction (Supplementary Figure S6B). We also observed that the poisoning effect could not be removed by higher centrifugation speeds. Syn3A lysates prepared by French press and clarified at different centrifugation speeds (20–80 000 g) (Supplementary Figure S7) depleted the production of eGFP in the *E. coli*/mycoplasma mixed CFE system, as observed in Figure 2A. However, because calcium-mediated nuclease inhibition restored eGFP expression in *E. coli* CFE mixed with Syn3A lysate, we reasoned that the nuclease content in mycoplasma lysates was perhaps too high for mycoplasma CFE. We next explored procedures for removing these poisoning components from mycoplasma lysates.

### 3.3 Trypsinization of mycoplasma cells removed the poisoning effect and suggested surface nuclease activity

**Figure 3.**
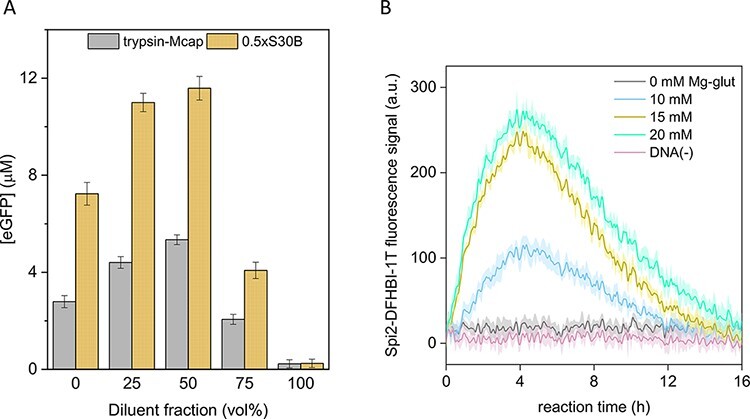
Effect of trypsinization of Mcap cells on the production of mRNA and eGFP protein in CFE reactions. (**A**) *E. coli* CFE of eGFP, mixed with trypsin-Mcap lysate or S30B buffer at different ratios. We observed a decrease in eGFP expression >75 vol% of either S30B buffer or trypsin-Mcap lysate. (**B**) Transcription of Spinach2 aptamer in trypsin-Mcap lysate. The fluorescence signal from the Spinach2-DFHBI-1T complex was detected, which indicated mRNA production. However, translation was not observed in CFE reaction containing 100% trypsin-Mcap lysate.

Because the extracellular mycoplasma surface could be a major source of nucleases that incapacitated the CFE of the extracts, we trypsinized mycoplasma cells before cell lysis to inactivate surface nucleases. Mcap lysate prepared from trypsinized cells (hereafter trypsin-Mcap) did not inactivate expression in the *E. coli* CFE platform (Figure 3A). Instead, the eGFP expression in *E. coli* CFE mixtures containing up to 50 vol% trypsin-Mcap lysate was similar to the eGFP expression observed for control experiments using S30B buffer as diluent. The increase in expression by adding up to 50 vol% of diluent into the *E. coli* CFE system indicates an improved expression of eGFP at lower concentrations of *E. coli* lysate, which might be due to the specific optimal volume fraction of lysate in the CFE reaction mix. Although trypsin-Mcap lysate did not inactivate *E. coli* CFE, translation remained disabled for the mycoplasma CFE platform containing 100% trypsin-Mcap lysate, even in the presence of calcium glutamate (0–20 mM) (Supplementary Figure S8). Only a small transcription level (Spinach2 aptamer) was detected (Figure 3B) compared to the signal obtained in the *E. coli* CFE system, which was at least 2-fold higher.

### 3.4 RNA degraded during mycoplasma lysate preparation

**Figure 4.**
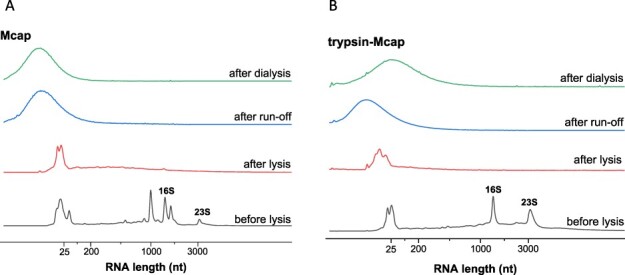
Integrity of native RNA in Mcap lysates prepared by sonication and centrifuged at 20 000 g. RNA profiles obtained from (**A**) Mcap and (**B**) trypsin treated Mcap cells (trypsin-Mcap) before lysis, after lysis, after run-off incubation, and after dialysis.

At this point, we reasoned that the development of a functional Mcap and/or Syn3A CFE was impeded by rapid degradation of RNAs by mycoplasma nucleases, leading to complete inactivation of the translational machinery early on during lysate preparation. Therefore, we decided to investigate the total RNA content at various stages of lysate preparation with the goal of understanding more about the lack of CFE. Analysis of the total RNA content by gel electrophoresis of several mycoplasma lysates revealed high degradation levels throughout the lysate preparation (Figure 4). Mcap lysate obtained by sonication and subsequent centrifugation at 20 000 g showed significant RNA degradation directly after cell lysis (Figure 4A, red line). Even the 16S and 23S rRNAs (Figure 4, black line), which were expected to be protected by ribosomal proteins, were greatly degraded after cell lysis. As the lysate preparation proceeded to run-off incubation and dialysis, RNA was completely degraded (Figure 4A, blue and green lines, respectively). Trypsin-Mcap lysate also degraded rRNA (Figure 4B), which suggested that trypsinization was insufficient to deplete all surface RNases from Mcap cells. We performed a similar analysis for Syn3A and trypsin-Syn3A lysates, but after lysis, RNA was found completely degraded as well.

### 3.5 Surface nucleases degraded foreign RNA

**Figure 5.**
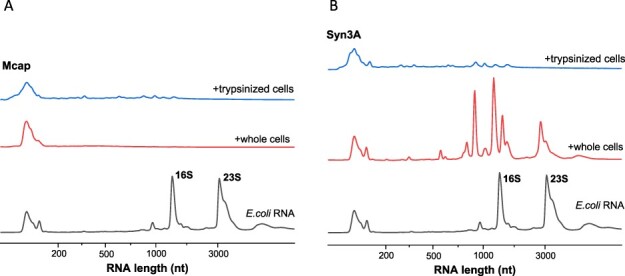
RNA degradation induced by surface RNases of (**A**) Mcap and (**B**) Syn3A cells. The control sample (bottom data line in panels A and B) shows intact RNA isolated from *E. coli* lysate. Addition of mycoplasma cells (middle data line in both panels SA and B) and trypsinized mycoplasma cells (top data line in both panels SA and B), caused *E. coli* rRNA to degrade into fragments <1500 nt. Compared to Mcap, Syn3A cells presented less active surface RNases.

To determine whether the mycoplasma cell membrane and associated proteins were the sources of the RNase activity, we assessed the degradation of RNA by whole mycoplasma cells using total RNA from *E. coli* lysate as a target. Whole Mcap or Syn3A cells were first separated from the culture medium using centrifugation through a sucrose cushion (0.5 M sucrose in 75 mM HEPES pH 8). During incubation at 37°C for 60 min, both purified Mcap and Syn3A cells degraded the longer rRNAs from *E. coli* into fragments smaller than 1500 bases (Figure 5, red lines), indicating RNase activity of the cell surface or exterior. A control experiment shows that rRNAs from *E. coli* lysate incubated at 37°C remain relatively stable within the same time scale (Supplementary Figure S9A). Considering that both Mcap and Syn3A were incubated under similar conditions, Syn3A cells showed lower RNase activity as 16S and 23S bands remained visible, whereas the same bands were absent with Mcap cell suspensions (under same incubation conditions). This result suggested rRNA degradation caused by nucleases present at the cell surface of Mcap and Syn3A. The degradation of rRNA from *E. coli* lysate was enhanced when incubated with mycoplasma lysates (Supplementary Figure S9C–J).

Cell trypsinization was insufficient to deplete RNase activity; whole trypsin-Mcap and trypsin-Syn3A cells purified by sucrose cushion still degraded RNA (Figure 5, blue lines). As observed for purified RNA aptamers (Supplementary Figure S5) and endogenous Mcap and trypsin-Mcap RNAs (Figure 4), mycoplasma cells rapidly degraded foreign *E. coli* RNA, whether cells were trypsinized or not. Also, the cell culture supernatant degraded total RNA from *E. coli*, confirming RNase activity from released nucleases (Supplementary Figure S9B, green line). Our controls, a 0.5-M sucrose solution and a fresh mycoplasma growth medium did not degrade *E. coli* RNA (Supplementary Figure S9B, blue and red lines, respectively).

Considering the unique biological features of mycoplasmas, the development of its CFE system will require new methods, especially for cell lysis and lysate clarification. We next tested cell disruption methods that are unusual for *E. coli* or *Bacilli* CFE systems.

### 3.6 Lysis by digitonin improved rRNA content in Mcap and Syn3A lysates

All cell disruption methods tested thus far were based on successful strategies to prepare *E. coli* or *Bacilli* CFE systems. Considering the unique biological features of mycoplasmas, we investigated alternative methods for cell lysis and lysate clarification. As mycoplasmas do not have a cell wall, but rather only a cholesterol-rich membrane, we explored milder disruption methods using detergents to solubilize the mycoplasma membranes. TX100 is a non-ionic surfactant previously used for membrane solubilization of *M. laidlawii* and *M. mobile* (54, 55). When we used TX100 to solubilize Mcap membranes (TX100-Mcap lysate), a small amount of 16S rRNA was observed, whereas 23S rRNA was completely degraded (Supplementary Figure S10, red line). Although the TX100-Mcap lysate had a slightly higher rRNA content than lysates prepared by sonication or French press, the lysate still did not support translation.

**Figure 6.**
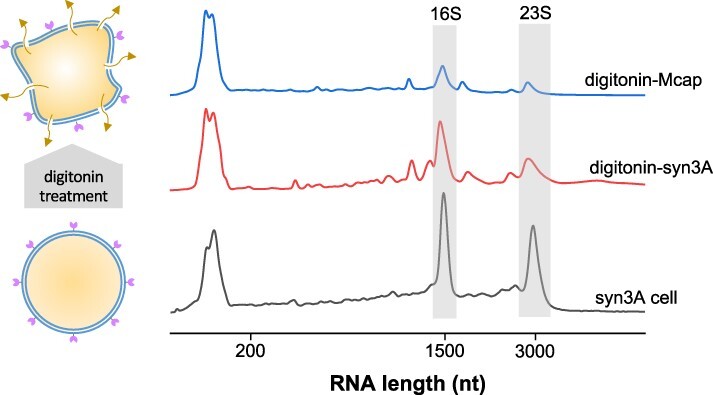
Digitonin permeabilizes the mycoplasma cell membrane, allowing cytosol to leak (left). RNA in lysates prepared from mycoplasma cells treated with digitonin (right). Bottom data line: RNA isolated from Syn3A cells before lysis. Syn3A cells yielded lysates with higher rRNA content (middle data line) compared to Mcap lysate (top data line).

Next, we tested digitonin, a detergent used for the lysis of mycoplasma cells (56–58) and subcellular fractionation of eukaryotic cells (59). Whereas TX100 completely solubilizes membranes, digitonin permeabilizes the membrane by precipitating cholesterol present in the mycoplasma membrane. Remarkably, lysates prepared from digitonin-treated Mcap cells (digitonin-Mcap) showed a higher content of rRNA compared to any other mycoplasma lysate (Figure 6, blue line). Apparently, purification of the extracted lysate from the larger permeabilized membranes was more effective in reducing RNase activity of the lysate. Syn3A cells treated with digitonin (digitonin-Syn3A) (Figure 6, red line) showed an even higher rRNA content than digitonin-Mcap, possibly due to the absence of some unidentified RNases. However, despite the higher rRNA content, digitonin-Syn3A lysate still failed to support CFE, even in the presence of calcium chloride as a nuclease inhibitor (Supplementary Figure S11).

Altogether, our results clearly show extensive nuclease activity, possibly originating from the interior as well as the exterior of Mcap and Syn3A, which apparently is detrimental for developing a robust mycoplasma CFE system, although perhaps not the only impediment.

### 3.7 Lysates prepared by digitonin treatment show reduced RNase activity

**Figure 7.**
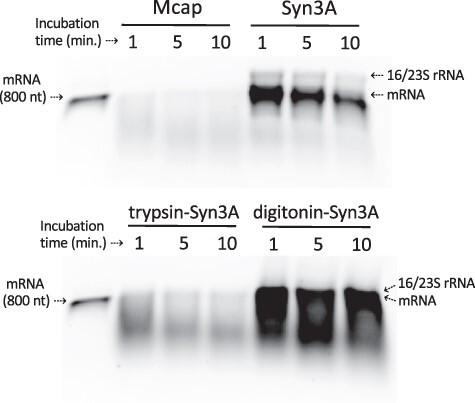
Comparative RNase activity among several mycoplasma lysates confirmed a higher RNase activity in Mcap lysate compared to Syn3A. The mRNA substrate (1.5 µg) was incubated at 30°C with mycoplasma lysate (protein concentration ∼3 mg/ml) for 1–10 min. RNA samples were analyzed using denaturing 1% agarose gel electrophoresis. The high rRNA content in the digitonin-Syn3A lysate suggested the presence of intact ribosomes. Mcap, Syn3A and trypsin-Syn3A lysates were prepared by sonication and digitonin-Syn3A lysate by digitonin treatment.

To compare the RNase activity among different mycoplasma lysates, we followed the degradation of an mRNA substrate over time. A denaturing agarose gel (Figure 7) showed rapid RNA degradation in Mcap lysate, followed by trypsin-Syn3A lysate and Syn3A lysate obtained by sonication. These results correlate with our previous observations regarding RNase activity in mycoplasma lysates (Figure 5). The mRNA degradation by digitonin-Syn3A lysate could not be observed directly in this experiment because of overlap with abundant rRNAs. However, although the mRNA band overlapped with the rRNA bands, its presence indicated much lower RNase activity in digitonin-Syn3A lysate. Further analysis of the RNA degradation by digitonin-Syn3A lysate compared with *E. coli* lysate confirmed reduced degradation of mRNA and rRNA in digitonin-Syn3A lysate (Supplementary Figure S12A). Despite these improvements, digitonin-derived lysates remained nonfunctional for eGFP production (Supplementary Figure S12B). Also, only a small level of transcription similar to the trypsin-Mcap lysate could be detected (Supplementary Figure S12C;Figure 3B).

## 4. Discussion

Features of mycoplasmas in general, while ideal for constructing synthetic minimal cells to study cellular life processes, may contribute to the pitfalls in developing CFE systems based on how such systems were developed for *E. coli* or other eubacteria. Our plan to construct a living synthetic cell from nonliving parts is dependent on developing CFE systems derived from mycoplasma bacteria. Discoveries about the limitations of the genome transplantation technique, where a synthetic genome from one species of bacteria is installed in a bacterial cell of a different species to create a new cell with the genotype and phenotype of the synthetic genome, convinced us that to construct a synthetic cell by booting up a mycoplasma genome we would need to use a CFE system from a closely related mycoplasma species (60). While we expected difficulties in our program to produce a synthetic cell, we did not expect producing a mycoplasma CFE system to be problematic. CFE systems have been created for a broad variety of Gram-positive and Gram-negative bacteria (61), and there are no literature reports describing bacterial species for which no CFE could be developed. In retrospect, we should have been more cognizant that it took many years to develop today’s efficient CFE systems based on *E. coli* and that CFE systems using bacteria other than *E. coli* produce only a small fraction of the amounts of proteins that can be obtained using *E. coli* CFE (18).

The methods developed to make effective CFE systems for *E. coli* and other bacteria, when applied to both Mcap and near-minimal bacterium Syn3A, resulted in cytoplasmic extracts in which we were unable to produce useful amounts of *in vitro* transcribed RNA. Our data indicate that mycoplasma RNase activity is at least one of the causes of this failure, but they do not rule out the possibility that there could be other factors confounding the development of a mycoplasma CFE. In an attempt to understand this, we looked at the principal differences between the biology of conventional bacteria, such as *E. coli* or *B. subtilis*, and the mycoplasmas. In our view, there are three salient differences:

- First, mycoplasmas cannot synthesize DNA or RNA precursors. Thus, they must import bases, nucleosides or and nucleotides. All of which are included in the mycoplasma growth media, but in nature where mycoplasmas are typically respiratory and urogenital parasites of mammals, reptiles, birds and fish, these molecules must be gleaned from all available sources to support mycoplasma growth. Nucleases that degrade nucleic acids to molecules more easily imported could be critical for mycoplasma survival in nature (although genes encoding such nucleases would likely have been nonessential for laboratory growth and thus not included in the minimal cell genome). The existence of membrane-associated RNase activity for several mycoplasma species is documented (47–52, 62, 63).
- Second, mycoplasma cells are much smaller than most other bacteria. The volume of a 1.5-µm long *E. coli* cell is ∼40 times greater than the volume of a typical 400-nm diameter Mcap cell. These size differences lead to a variety of physiological differences. Notably, since ribosome and protein concentrations systematically shift with cell size, the ratio of these two macromolecules varies by a factor of three between mycoplasmas and *E. coli* (64). Similarly, smaller cells have much higher surface-to-volume ratios than larger cells affecting the flux of nutrients to the cell and the requirements for transporters (65). These drastic differences in ratios and macromolecular requirements may introduce novel tuning considerations for mycoplasmas in how to properly adjust their concentrations of various macromolecules. Similarly, the lifestyle and size of mycoplasma likely imply sensitivity and lack of robustness to noise for two reasons. First, as obligate parasites mycoplasmas often experience the homeostatic environment of their host compared with the large and chemically diverse environmental fluctuations experienced by many other bacteria. In fact, genome reduction is often argued to be associated with more consistent environments (66, 67). Second, the cellular environment of mycoplasmas is defined by truly discrete abundances of many of the macromolecules, and this may mean that typical cellular physiology is defined by precisely tuned concentrations compared with larger bacteria. This may mean that the physiological and regulatory dynamics of mycoplasmas may be evolved to precisely regulate certain abundances, and it may be the case that CFE systems are not precise enough to capture this tuning.
- Third and perhaps most important, mycoplasmas have no cell walls. Cells are enveloped by phospholipid bilayer membranes containing cholesterol. The methods used to produce CFE for conventional bacteria, such as sonication, French press or digitonin treatment followed by centrifugation to separate cytoplasm from the cell envelope, may not work for cells lacking cell walls. We will investigate whether the CFE system methods used to purify the cytoplasm from conventional bacteria that have cell walls are not effective at eliminating membrane-bound nuclease activity in bacterial cells, where the lipid bilayer membrane is not tethered to the cell wall. The 30–34 000 g, 10–15-min centrifugation step on which S30 extracts are named that we used to separate cytoplasm from cell envelope may not be working. RNases associated with membrane fragments generated by digitonin treatment or French press may not be spun away from the cytoplasm during the centrifugation.

Each of these mycoplasma attributes could have contributed to the presence of nuclease activity in our efforts to make mycoplasma CFE systems. As for the actual Syn3A gene product(s) responsible for the problematic RNase activity, we know it was present on the surfaces of cells. This was because intact mycoplasma cells were capable of poisoning *E. coli* CFE systems. Cell surface-associated RNase activity correlated with the mycoplasma need to scavenge and import external nucleotides. It was noteworthy that Syn3A, which contains a subset of the nuclease-encoding genes present in Mcap, was capable of supporting better *in vitro* transcription than Mcap (Supplementary Figure S5). Furthermore, lysates prepared by digitonin treatment of Syn3A cells presented the lowest RNase activity among all mycoplasma lysates prepared in this study. This suggested to us that at least one or more nuclease-encoding genes were deleted as a result of genome minimization.

Armed with data showing the nuclease activity was membrane-associated, we looked carefully at the only two annotated Mcap and Syn3A genes that encode membrane-associated RNases (Supplementary Table S2). RNase Y is part of the degradosome complex and is highly abundant in the cell (53, 68). The other gene, provisionally annotated as RNase HII, is involved in the degradation of the ribonucleotide moiety on RNA-DNA hybrid molecules carrying out endonucleolytic cleavage to 5′-phospo-monoester (69). However, that gene, which is not an exact match to characterized RNase HII enzymes, may have evolved in mycoplasmas for a different purpose. Still, in Syn3A, there are fewer than 10 copies of the putative RNase HII per cell (5). Because both of these potential RNases are essential, it is not practical to genetically engineer away their nuclease activity. Because the RNase activity was so potent, we hypothesize the likely issue is the degradosome, which is an enzymatic complex comprising multiple RNases that is responsible for recycling most of the RNA cells, and even in a tiny Syn3A cell, there are hundreds of degradosome complexes (53). Alternatively, the source of the RNase activity causing our problems may be one of the >90 Syn3A genes of unknown function; however, none of those genes have domains suggesting RNase activity. Even though nucleases seem to be a major cause of the mycoplasma CFE inefficacy, the existence of other issues cannot be completely ruled out.

The attempts to produce a mycoplasma lysate using surfactants point out the importance of (i) maintaining the overall structure of the membrane during cell lysis and (ii) quickly removing cellular debris by centrifugation. Those principles will lead to a lower level of membrane shearing or solubilization leads, which means a lower release of surface nucleases into the final lysate. Also, milder cell fragmentation with digitonin treatment could have allowed more efficient removal of cellular debris by centrifugation. The high energy employed for cell disruption in sonication and French press methods may be one of the reasons for high nuclease activity. Another way to deplete nucleases in the lysate would be by down-regulating or knocking out nuclease-related genes in mycoplasma cells. This is however complicated for mycoplasma because several of the annotated nucleases are either essential or quasi-essential genes, which poses additional challenges to obtain such mutants.

As an alternative to mycoplasma lysate-based CFE systems, mycoplasma DNA (codon-optimized for mycoplasma usage (70)) could be used to produce the enzymes needed to construct a reconstituted cell-free protein expression system. Referred to as PURE systems (2), this technology might be adapted to use mycoplasma enzymes. This approach would help circumvent the nucleases derived from the mycoplasma cell during lysate preparation. Since PURE technology was initially designed as an *E. coli* cell-free system, a PURE system for mycoplasma would likely require considerable fine-tuning.

Of course, it may be that despite the attractiveness of mycoplasmas as chasses for the construction of synthetic cells, this problem of creating CFE systems with mycoplasmas is insurmountable. Efforts to construct a synthetic bacterial cell from nonliving parts may need to use RNA and protein expression systems comprised of materials obtained from a more conventional bacterium. The observation in genome transplantation that the donor genome must come from a species very closely related to the recipient cell suggests that we could not boot up a mycoplasma genome using a CFE from a conventional bacterium (60). So, while we search for either a genetically altered mycoplasma strain absent the RNase activity or a preparation technique that will produce an effective mycoplasma CFE system, we will also consider using a genome and CFE from a conventional small genome bacterium, such as *Lactococcus lactis*, as a chassis for the construction of a synthetic cell from nonliving parts.

## 5. Conclusion

While our attempts to produce a functional mycoplasma lysate described here were unsuccessful, it is important to consider that >40 years of research was necessary to produce a robust and typically high-yielding *E. coli* CFE system (1, 71). With that in mind, our team or another may still be able to overcome the technical challenges we describe here in order to produce a functional CFE system based on mycoplasmas.

## Supplementary Material

ysac008_Supp

## Data Availability

All bacterial strains and non-commercially available materials described in this paper are available to qualified researchers after completion of a material transfer agreement. A template for the material transfer agreement ‘JCVI-CodexDNA MTA for minimal cell.template.docx’ is included in the Supplementary Material.
