# Supplementary material for: Traditional protocols and optimization methods lead to absent expression in a mycoplasma cell-free gene expression platform
Source: Synth Biol (Oxf). 2022 May 21;7(1):ysac008. doi: 10.1093/synbio/ysac008 (PMC9239315; doi:10.1093/synbio/ysac008)
Supplement: ysac008_Supp [file ysac008_supp.zip › 20220408 SI.docx]

**Traditional protocols and optimization methods lead to absent expression in mycoplasma cell-free gene expression platform**

**Supplementary information**

| **(A)**  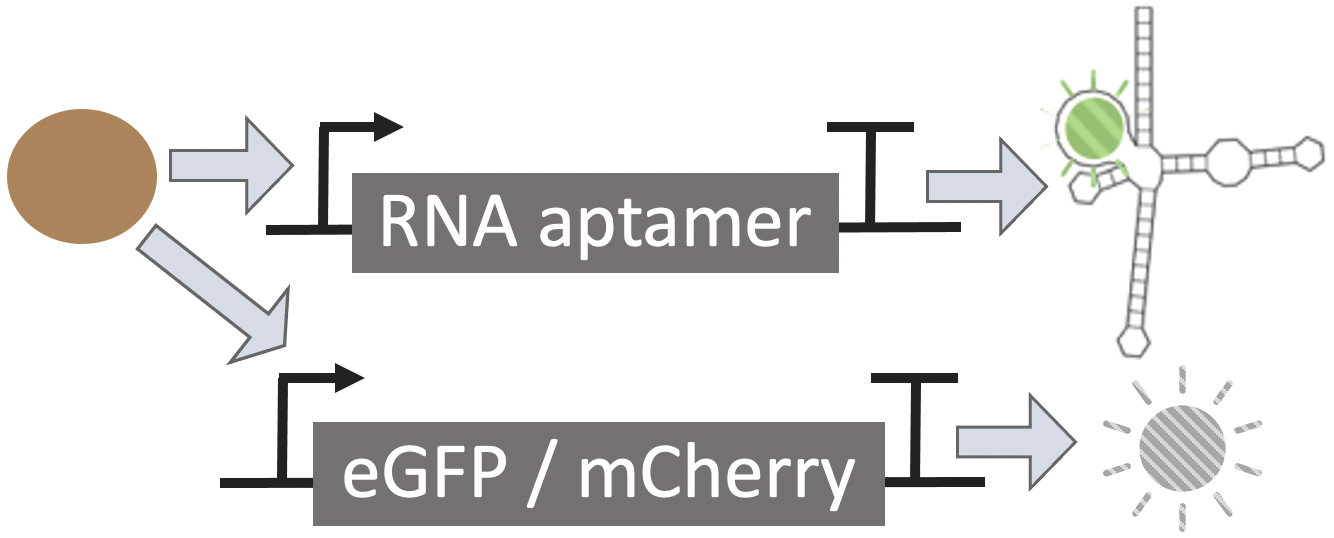 |  |
| --- | --- |
| **(B)**  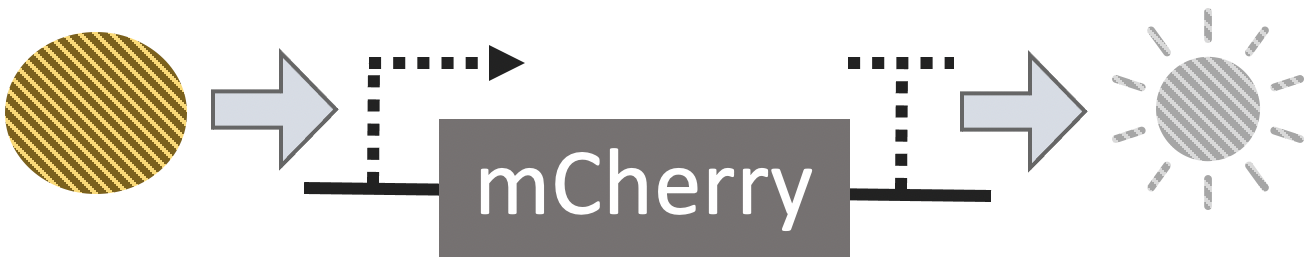 | 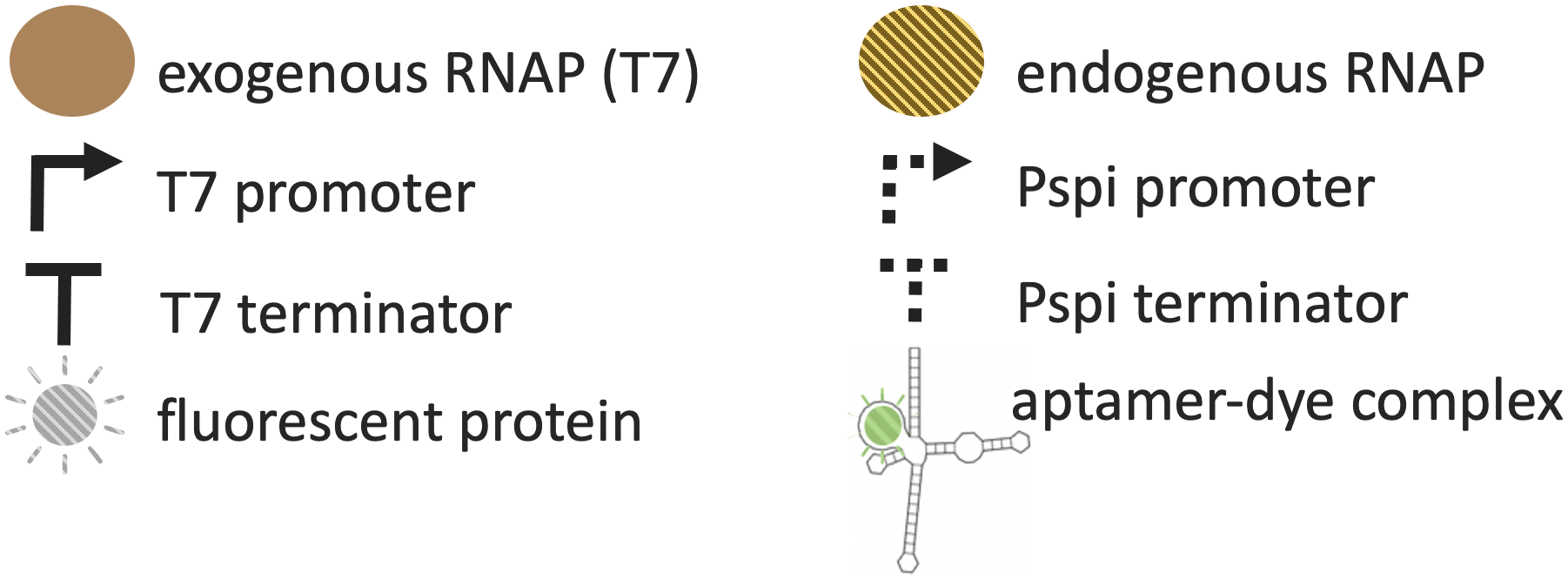 |

**Figure S1.** DNA constructs for testing expression in mycoplasma CFE system. **(A)** All coding sequences for RNA aptamers and fluorescent proteins were flanked by an upstream T7 promoter (sequence in Table S5) and a downstream T7 terminator. T7 RNA polymerase (T7 RNAP) was purified from *E. coli* and added to the CFE reaction for expression of T7-controlled genes. Green and red fluorescent proteins (eGFP/mCherry) were used to track translation. **(B)** The mCherry sequence codon-optimized for mycoplasma usage was provided by Prof. Yo Suzuki from the J. Craig Venter Institute (1). In the original DNA construct, mCherry gene is controlled by Pspi promoter and endogenous RNA polymerase (RNAP).

(D)

(C)

**
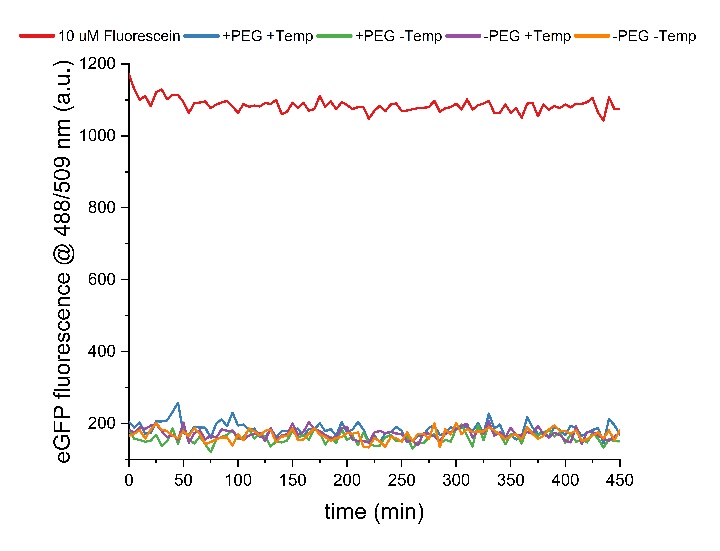
**

**(D)**

**(C)**

**(B)**

**(A)**

**
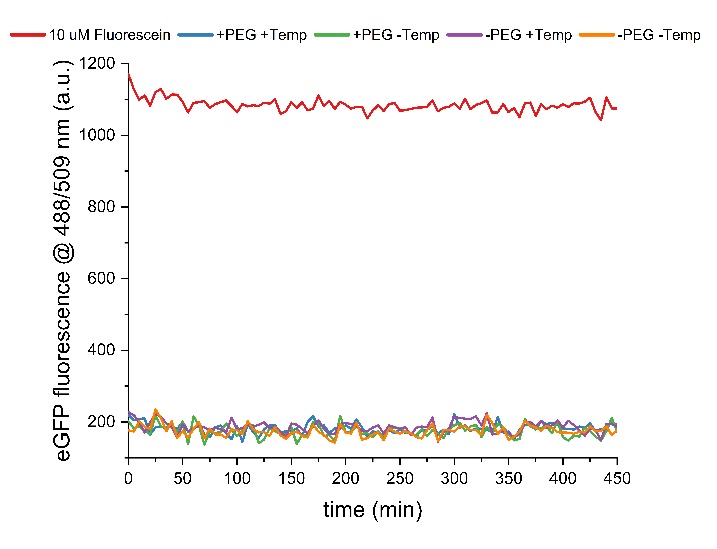
**

**
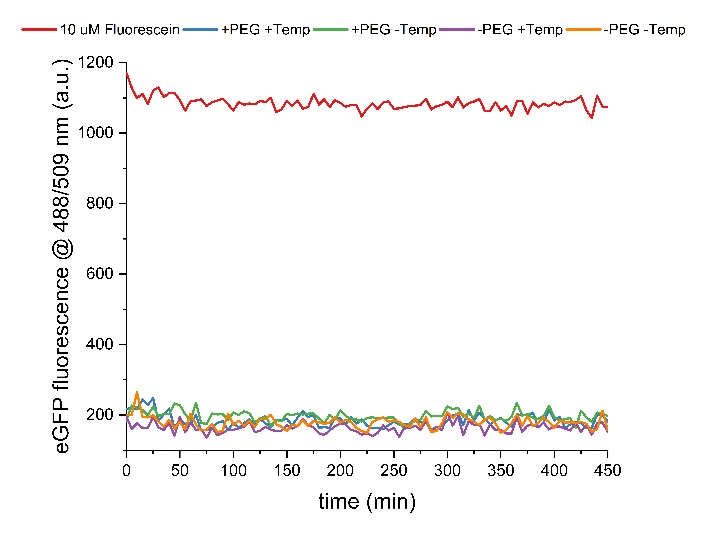
**

**
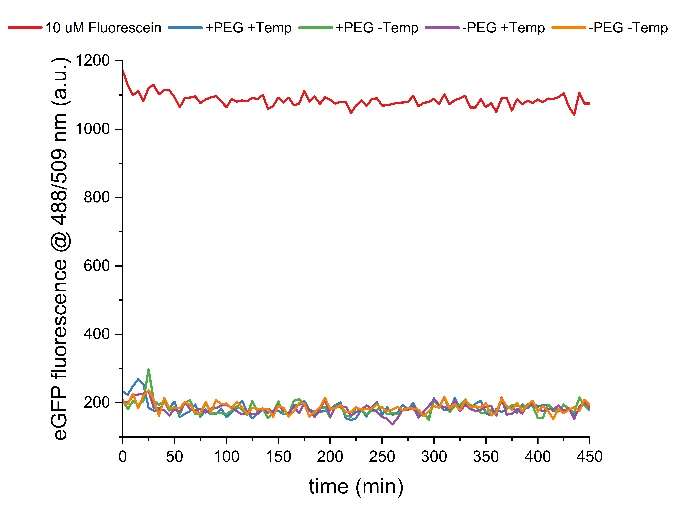
Figure S2. A-O Legend**

**
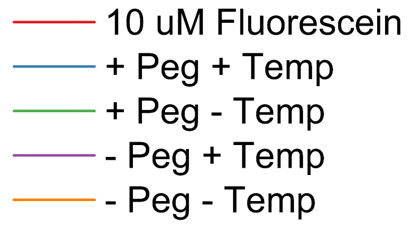
**

**Figure S2.** Effect of different lysate methods, energy source, run-off incubation and molecular crowder conditions on eGFP expression in mycoplasma-lysate CFE. Reactions were run with or without PEG800 (+/- PEG) and DNA template (+/- Temp). **(A)** 500 J sonication lysis of Mcap followed by 3000 g centrifugation, no run-off, addition of 2% PEG8000, and PEP. **(B)** 500 J sonication lysis of Mcap followed by 3000 g centrifugation, no run-off, addition of 2% PEG8000, and 3-PGA. **(C)** 500 J sonication lysis of Mcap followed by 3000 g centrifugation, run-off, addition of 2% PEG8000, and PEP. **(D)** 500 J sonication lysis of Mcap followed by 3000 g centrifugation, run-off, addition 2% of PEG8000, and 3-PGA.

(H)

(G)

**
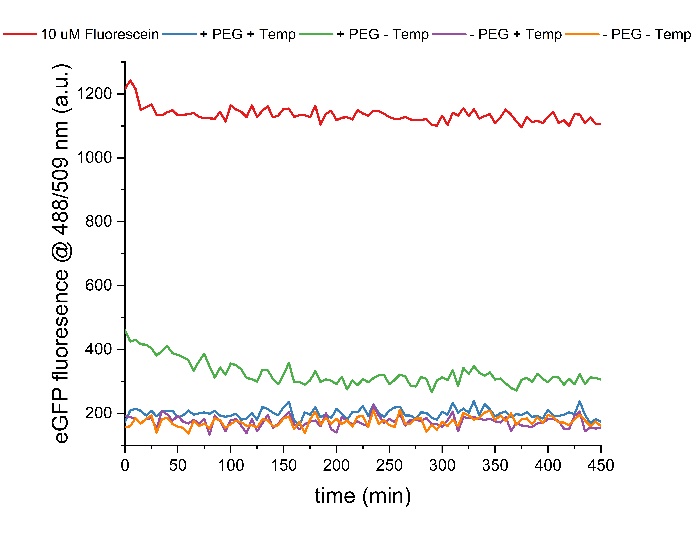

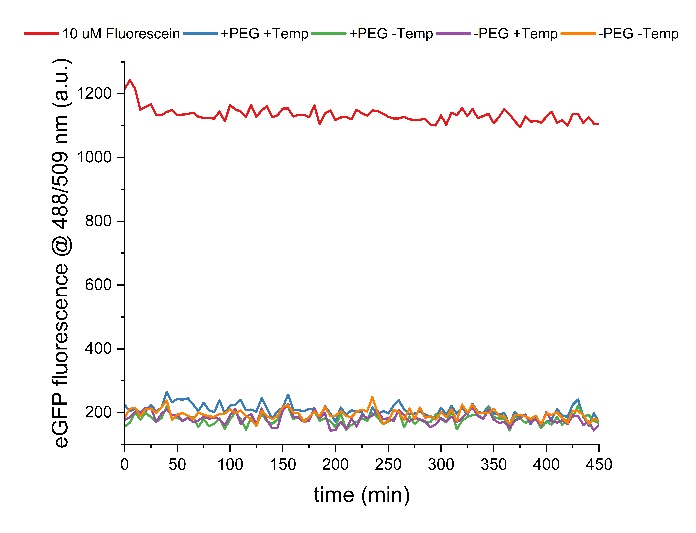
**

**(G)**

**(F)**

**(E)**

**
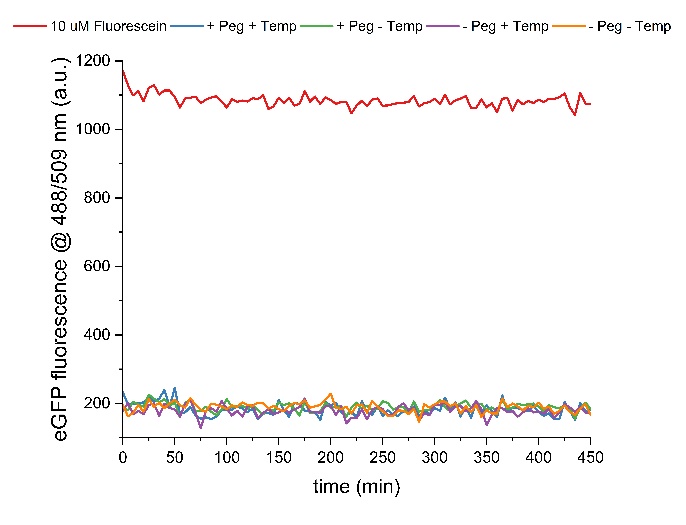

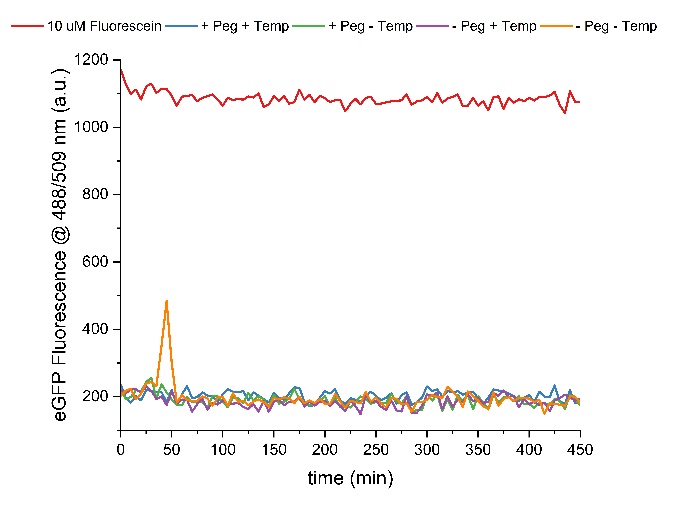
**

**(H)**

**Figure S2. Cont. (E)** 300 J sonication lysis of Mcap followed by 3000 g centrifugation, no run-off, addition of 2% PEG8000, and PEP. **(F)** 300 J sonication lysis of Mcap followed by 3000 g centrifugation, no run-off, addition of 2% PEG8000, and 3-PGA. **(G)** 300 J sonication lysis of Mcap followed by 3000 g centrifugation, run-off, addition of 2% PEG8000, and PEP. **(H)** 300 J sonication lysis of Mcap followed by 3000 g centrifugation, run-off, addition of 2% PEG8000, and 3-PGA.

**
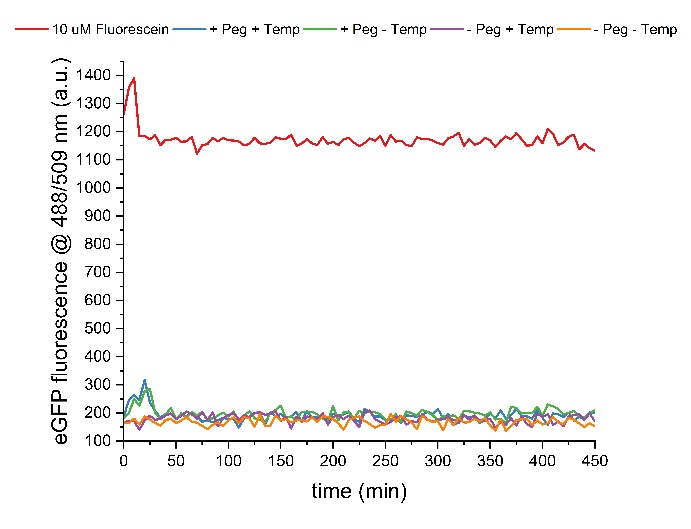

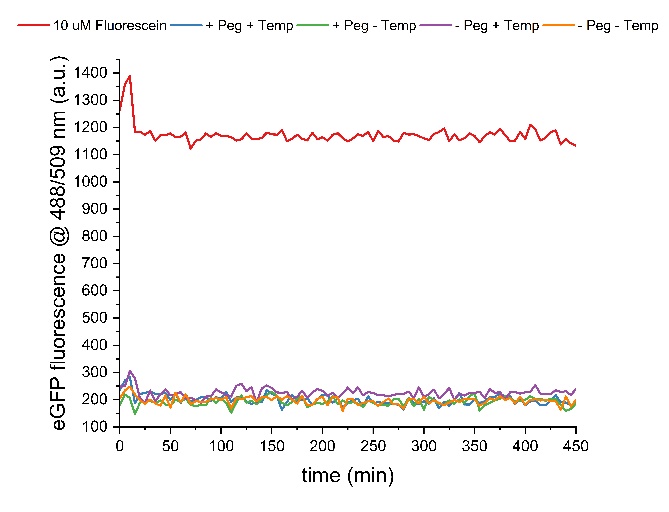

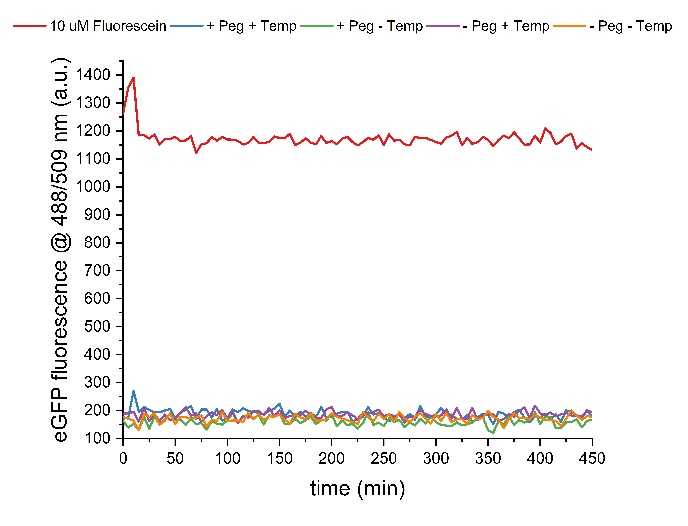

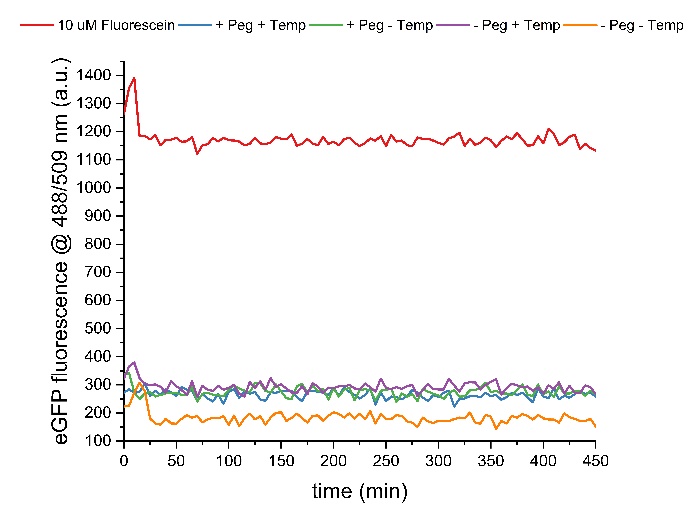
**

**(L)**

**(K)**

**(J)**

**(I)**

**Figure S2. Cont. (I)** 150 J sonication lysis of Mcap followed by 3000 g centrifugation, no run-off, addition of 2% PEG8000, and PEP. **(J)** 150 J sonication lysis of Mcap followed by 3000 g centrifugation, no run-off, addition of 2% PEG8000, and 3-PGA. **(K)** 150 J sonication lysis of Mcap followed by 3000 g centrifugation, run-off, addition of 2% PEG8000, and PEP. **(L)** 150 J sonication lysis of Mcap followed by 3000 g centrifugation, run-off, addition of 2% PEG8000, and 3-PGA.

**Figure S2. Cont.** **(M)** 500 J sonication lysis of Mcap followed by 12,000 g centrifugation, run-off, addition of 2% PEG8000, and 3-PGA. **(N)** 300 J sonication lysis of Mcap followed by 12,000 g centrifugation, run-off, addition of 2% PEG8000, and 3-PGA. **(O)** 150 J sonication lysis of Mcap followed by 12,000 g centrifugation, run-off, addition of 2% PEG8000, and 3-PGA**
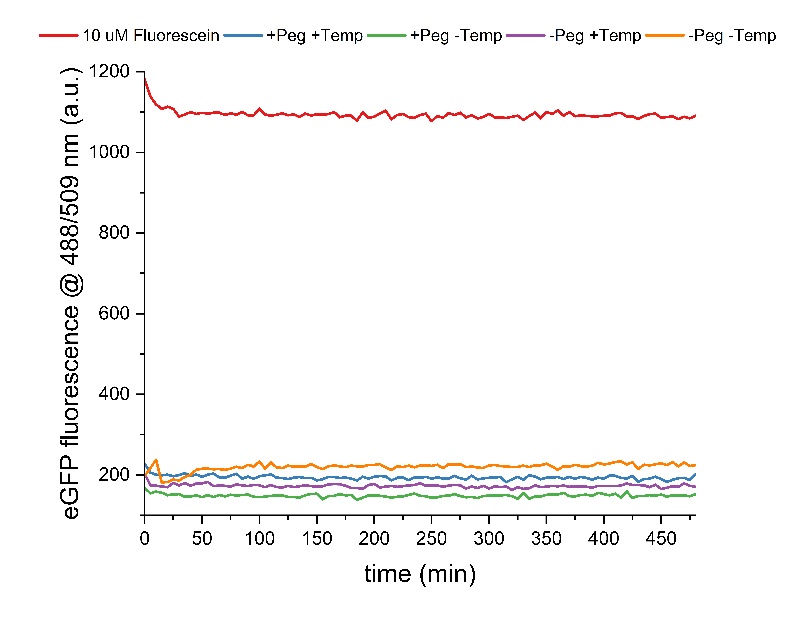
**.

**(M)**

**(N)**

**
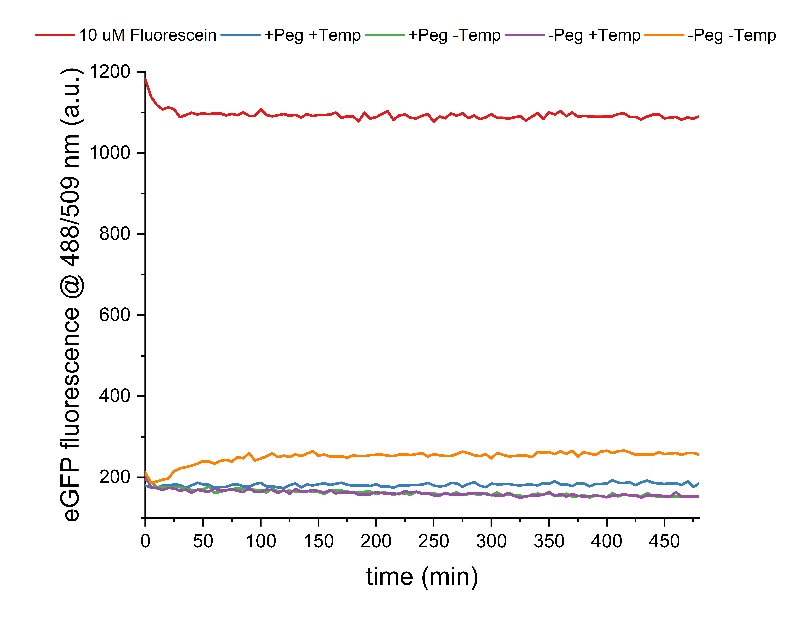
**

**(O)**

**
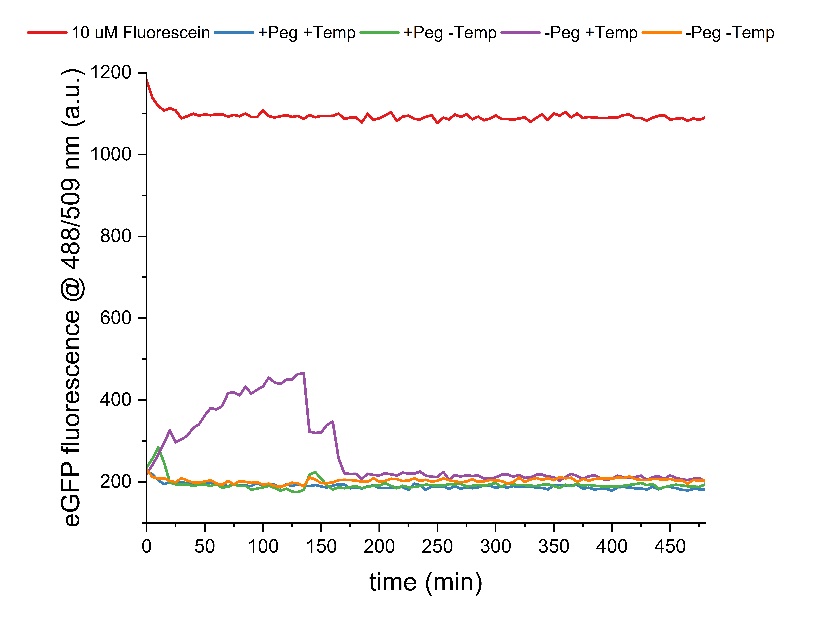
**

**(P)**

**
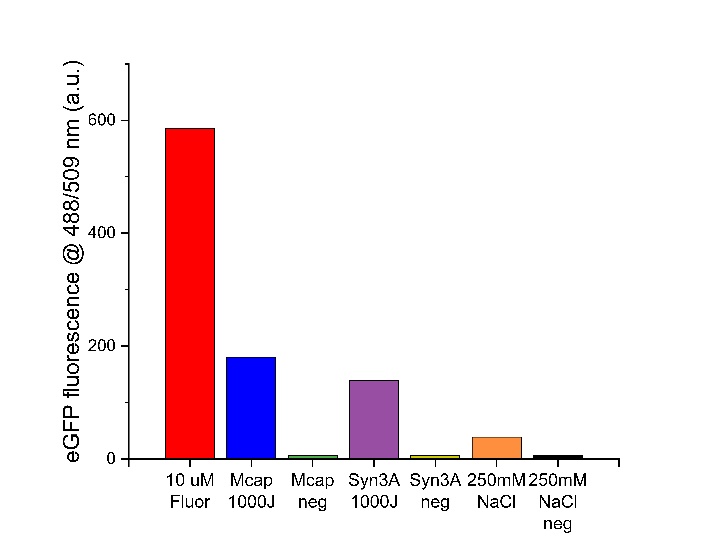
**

(Q)

**(Q)**

**
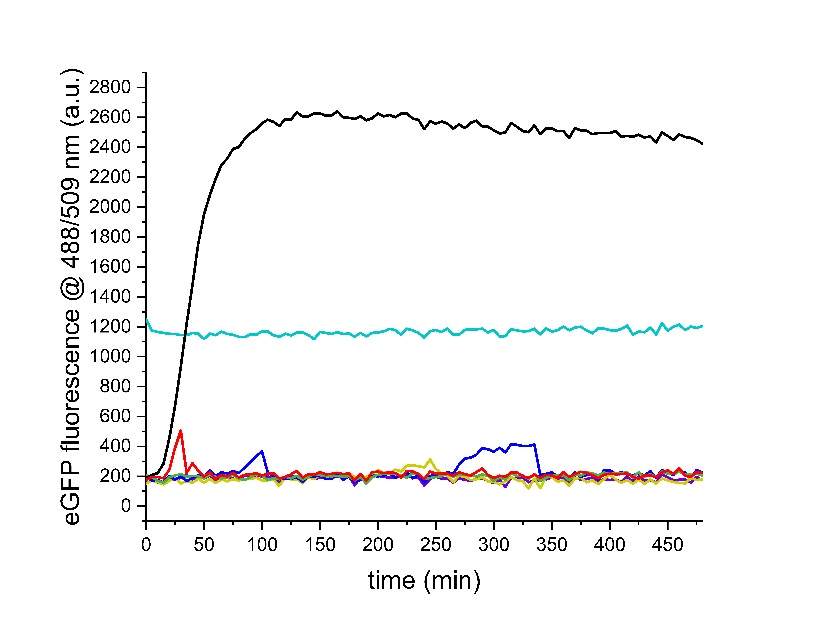
**

**
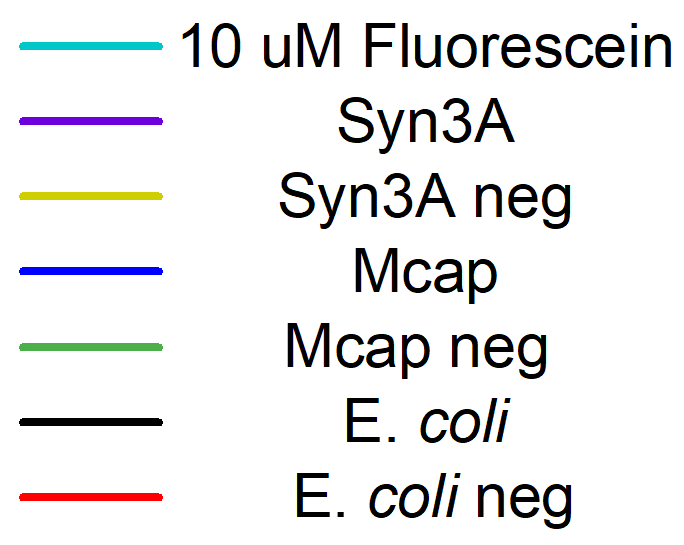
**

**Figure S2. Cont. (P)** 1000 J sonication lysis or 250 mM osmotic lysis of Mcap and Syn3A followed by 12,000 g centrifugation, run-off, addition of 3-PGA. **(Q)** Liquid nitrogen grind lysis of Mcap and Syn3A followed by 12,000 g centrifugation, run-off, addition of 3-PGA. Negative control reactions were run without DNA template.

**
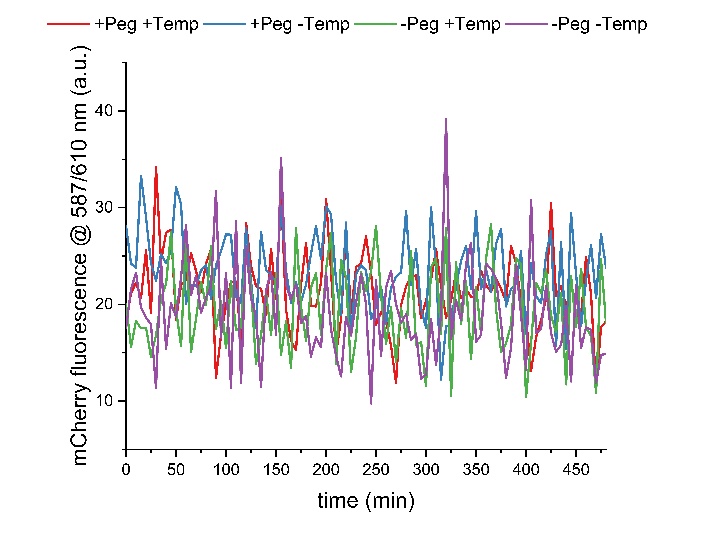

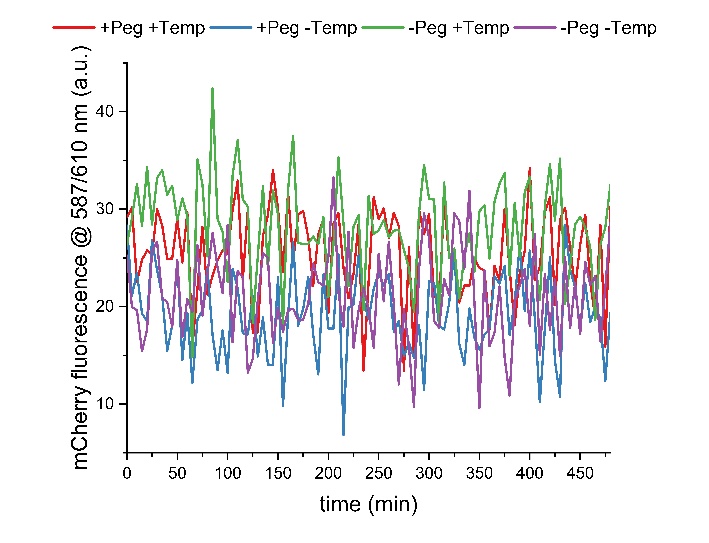
**

**(B)**

**(A)**

**(D)**

**(C)**

**
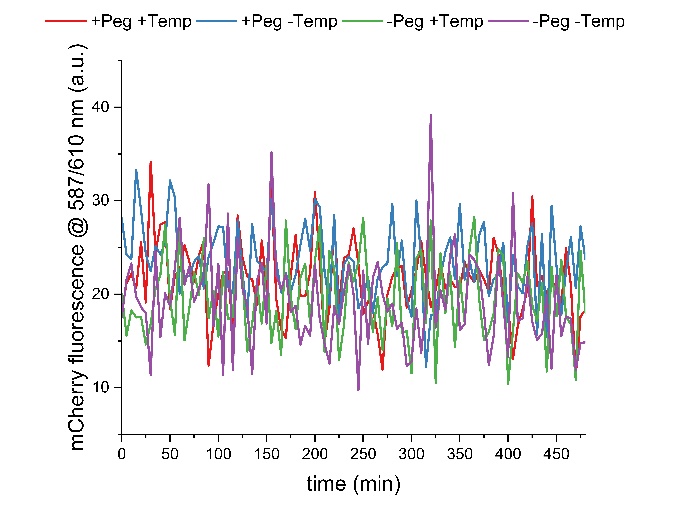

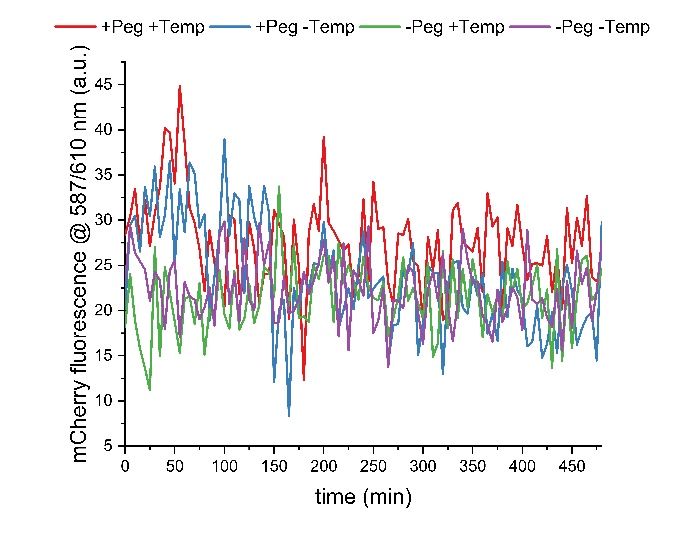
**

**Figure S3.** Effect of different lysate methods, energy source, run-off incubation, and molecular crowder conditions on mCherry expression in mycoplasma CFE under control of the Pspi promoter. Reactions were run with or without PEG800 (+/- PEG) and DNA template (+/- Temp). **(A)** 1000 J sonication lysis of Mcap followed by 12,000 g centrifugation, run-off, addition of 2% PEG8000 and 3-PGA. **(B)** 500 J sonication lysis of Mcap followed by 12,000 g centrifugation, run-off, addition of 2% PEG8000 and 3-PGA. **(C)** 300J sonication lysis of Mcap followed by 12,000 g centrifugation, run-off, addition of 2% PEG8000 and 3-PGA. **(D)** 250 mM NaCl osmotic lysis of Mcap followed by 12,000 g centrifugation, run-off, addition of 2% PEG8000 and 3-PGA.

**
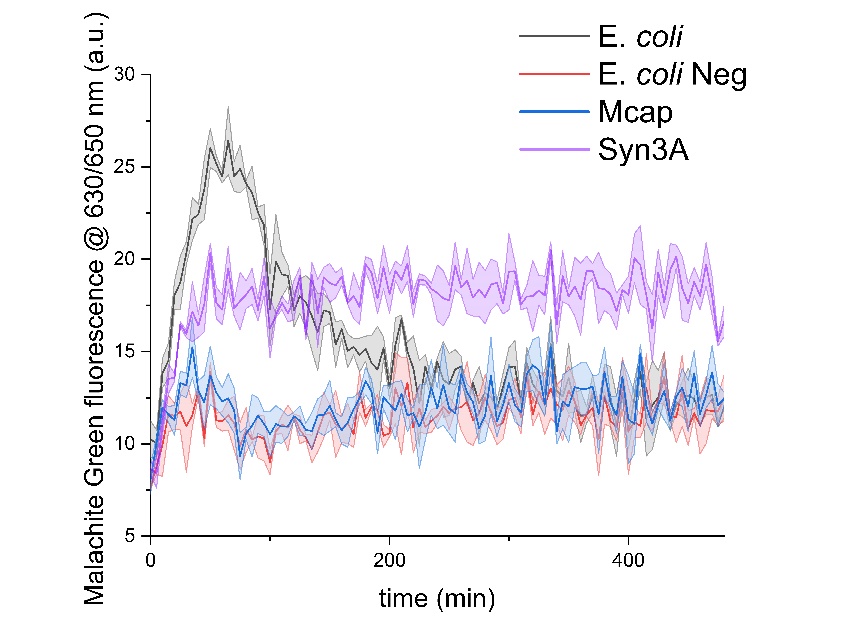
(A)**

**Figure S4.** Transcription of malachite green template in Mcap and Syn3A lysate prepared by liquid nitrogen grinding. *E. coli* lysate was included as positive control. Negative controls contained no DNA template. Shaded regions indicate standard deviation (n=3). **(A)** Direct comparison of malachite green fluorescence in positive template reactions.

**
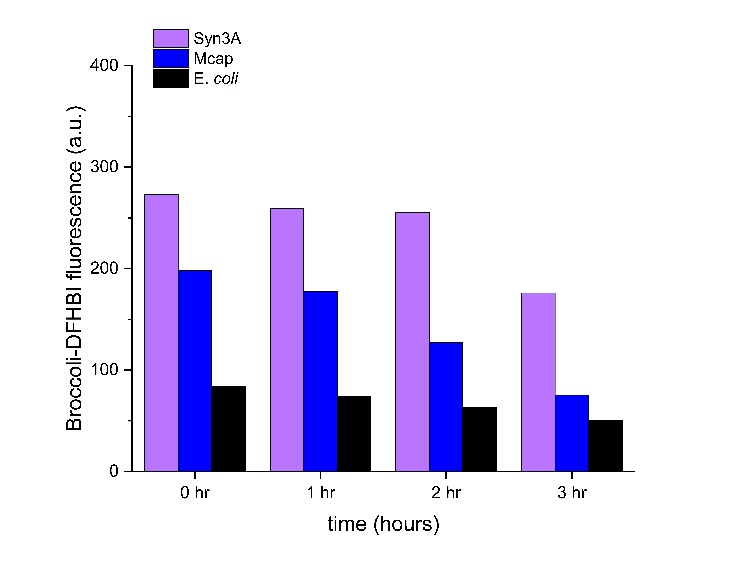
**
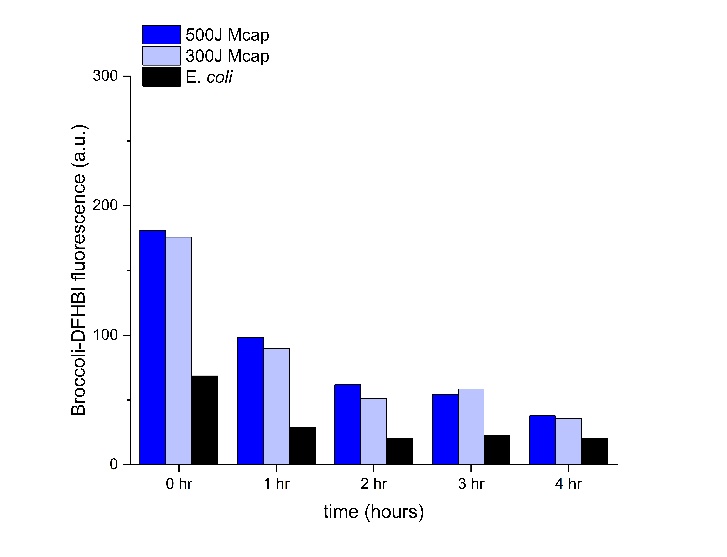


**(B)**

**(A)**

**(D)**

**(C)**

**
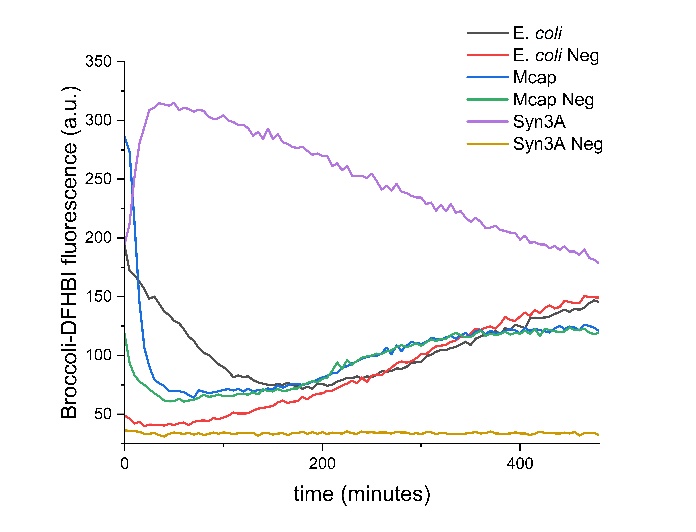
**

**
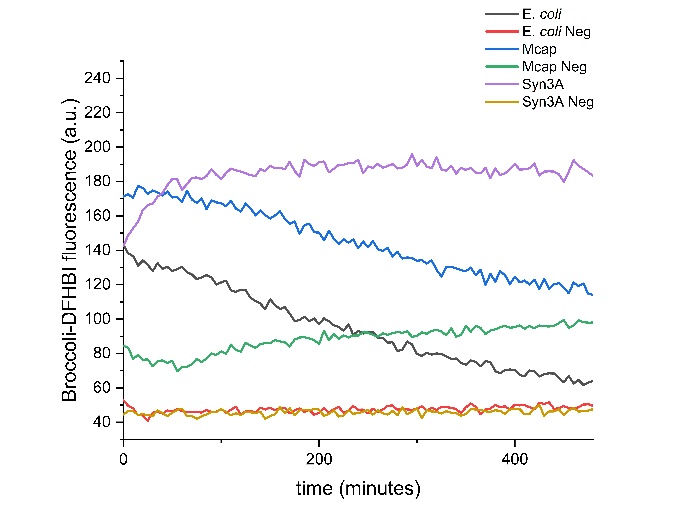
**

**Figure S5**. RNA degradation in Mcap and Syn3A lysate over time at 30˚C. Pre-expressed Broccoli aptamer was incubated with DFHBI ligand in folding buffer (1mM MgCl_2_, 50 mM KCl, Tris 10 mM pH 8) for 20 min at room temp before addition to lysates at a concentration of 1.25 µM. Broccoli fluorescence was read at 472/507 nm (a.u.). *E. coli* obtained by 500J lysis was used as a reference. Negative control reactions contained no Broccoli aptamer. **(A)** Stability of DFHBI-Broccoli complex in Mcap lysate obtained by sonication up to 500 J. **(B)** Stability of DFHBI-Broccoli complex in Mcap and Syn3A lysate obtained by sonication up to 300 J. **(C)** Kinetic assay of DFHBI-Broccoli stability in Syn3A and Mcap lysate obtained by sonication up to 300 J. **(D)** Kinetic assay of DFHBI-Broccoli stability in Syn3A and Mcap lysate obtained by liquid nitrogen grinding extraction.

| **(A)** | **(B)**   |
| --- | --- |

**Figure S6.** Effect of different calcium salts on eGFP production in mycoplasma CFE. Either calcium chloride or calcium acetate failed to restore gene expression. **(A)** CFE with 100% Syn3A lysate after 10 h for a range of 0 to 25 mM calcium chloride. **(B)** Using calcium acetate (0-15 mM) instead of calcium chloride did not show CFE of eGFP in either Mcap or Syn3A lysate. DNA(+): CFE with DNA template; DNA(-): CFE in absence of DNA template.

**Figure S7.** eGFP expression in *E. coli* CFE reactions mixed with different amounts of syn3A lysates prepared by French press and different centrifugal forces. All lysates disabled eGFP expression in *E. coli* CFE platform.

**
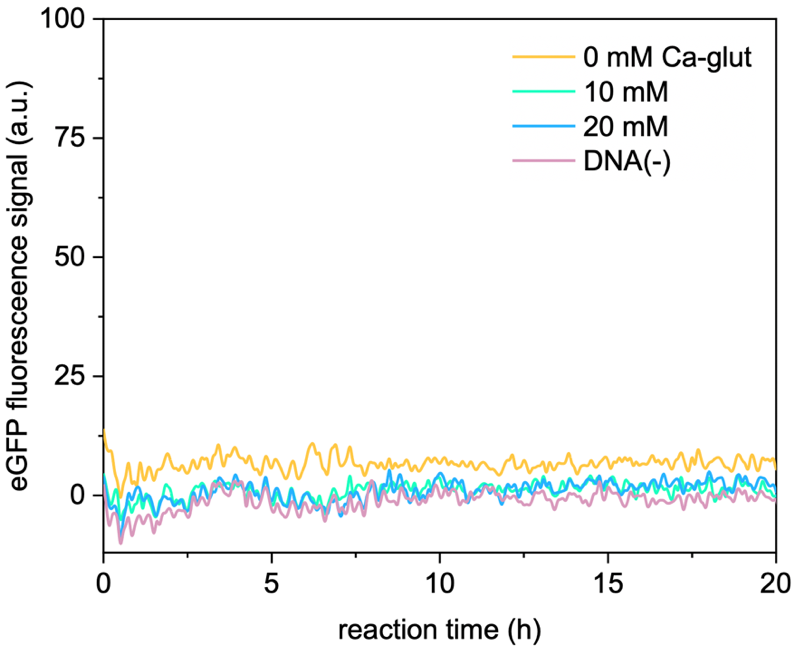
**

**Figure S8.** CFE reaction to produce eGFP protein in 100% trypsin-Mcap lysate in the presence of calcium glutamate. Expression of eGFP was not detected. DNA(-): control in absence of DNA template.

| **(A)**  **** | **(B)**  **** |
| --- | --- |
| **(C)**  *E. coli* rRNA + Mcap lysate  **** | **(D)**  *E. coli* rRNA + Mcap/Calcium lysate  **** |
| **(E)**  *E. coli* rRNA + trypsin-Mcap lysate  **** | **(F)**  *E. coli* rRNA + digitonin-Mcap lysate  **** |
| **(G)**  *E. coli* rRNA + Syn3A lysate  **** | **(H)**  *E. coli* rRNA + Syn3A/Calcium lysate  **** |
| **(I)**  *E. coli* rRNA + trypsin-Syn3A lysate  **** | **(J)**  *E. coli* rRNA + digitonin-Syn3A lysate  **** |

**Figure S9. (A)** Control experiment shows the stability of rRNAs in *E. coli* lysate during incubation at 37°C for 60 minutes. **(B)** Sucrose cushions (0.5M sucrose in 75 mM HEPES pH 8) were used to separate whole cells from culture medium. SP4 culture medium used for growing Syn3A degraded RNA, similar to Syn3A cells (green line). Controls demonstrated no ribonuclease activity in sucrose solution (blue line) or fresh SP4 culture medium (red line). Black line: intact RNA isolated from *E. coli* lysate. **(C-J)** *E. coli* lysate gets degraded within the first minute of incubation at 37°C with **(C)** Mcap lysate, **(D)** Mcap lysate with 20 mM calcium chloride, **(E)** trypsin-Mcap lysate, **(F)** digitonin-Mcap lysate, **(G)** Syn3A lysate, **(H)** Syn3A lysate with 20 mM calcium chloride, **(I)** trypsin-Syn3A lysate, **(J)** digitonin-Syn3A lysate.

**Figure S10.** RNA content in Mcap lysates obtained by treatment with surfactants. When TX100 was used for cell lysis (TX100-Mcap, red line), the final lysate showed a small amount of 16S rRNA while 23S rRNA was completely absent. The numerous peaks between 200 and 1,500 nt indicate a high level of rRNA degradation. When digitonin was used to permeabilize Mcap cells (digitonin-Mcap, blue line),a higher 23S rRNA content was observed. Additional steps combined with digitonin treatment, involving freeze-thaw (green line) or sonication(purple line), further increased RNA degradation.


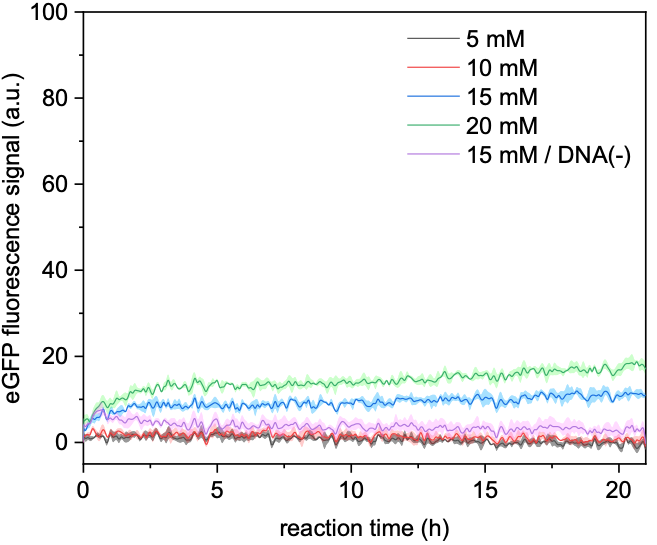


**Figure S11.** Expression of eGFP in mycoplasma CFE with digitonin-Syn3A lysate with various concentrations of calcium chloride. eGFP expression was not detected. DNA(-): control in absence of T7-eGFP plasmid DNA template.

| **(A)** |  |
| --- | --- |
| 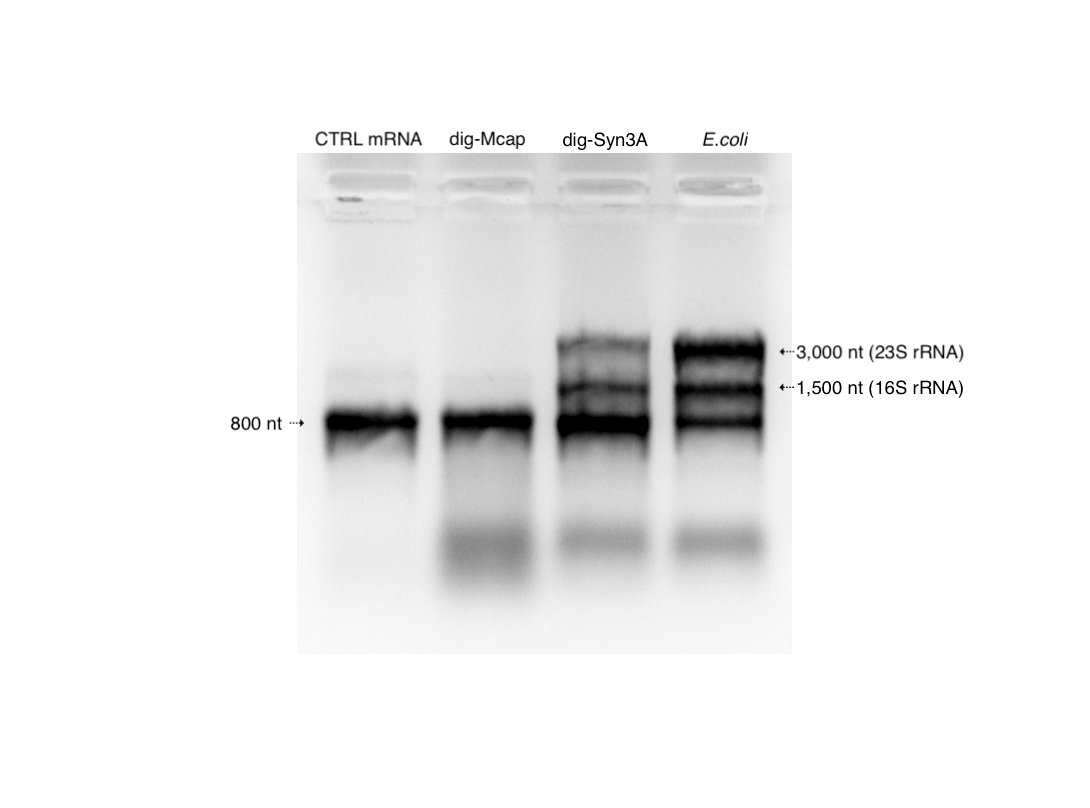 |  |
| **(B)** | **(C)** |
|  | 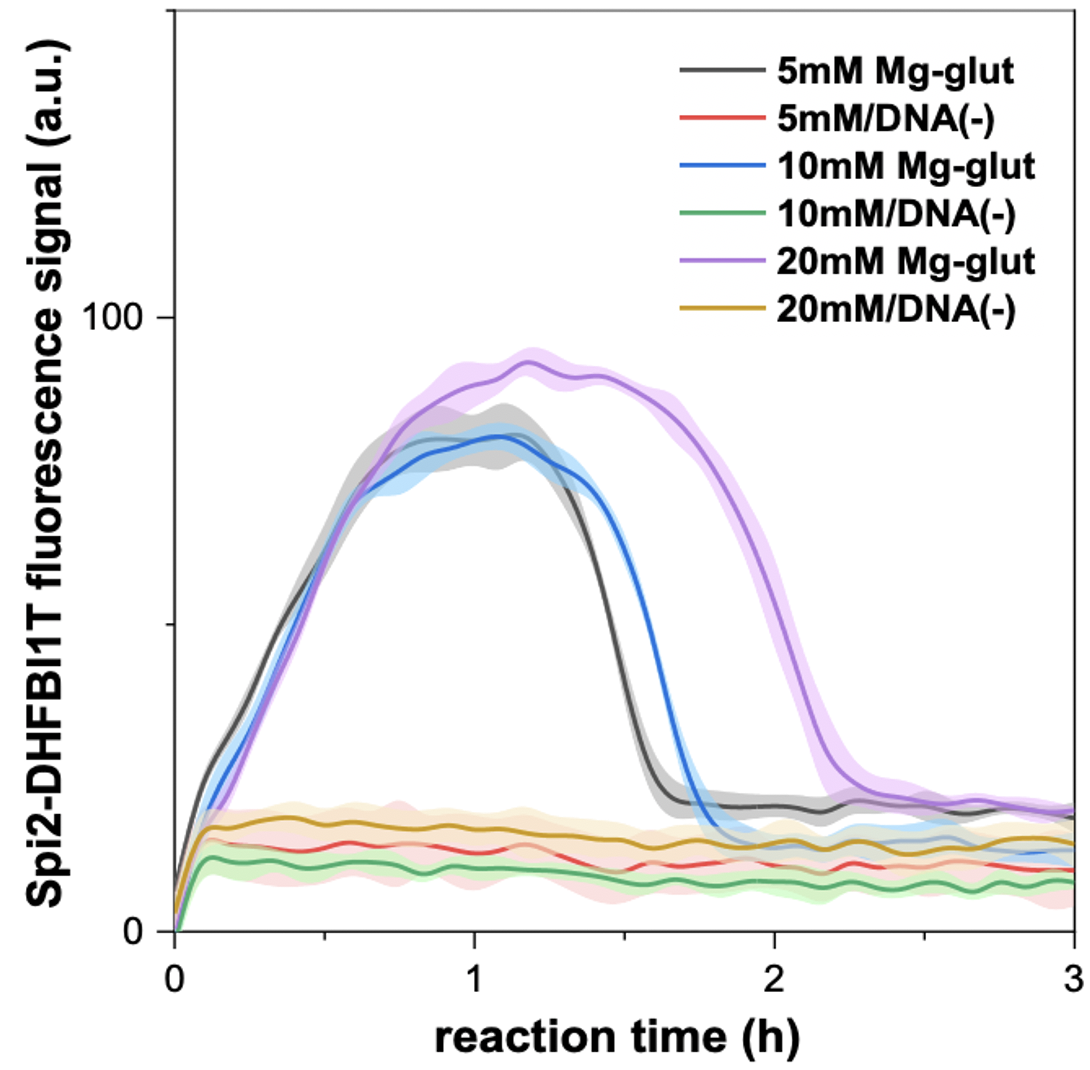 |

**Figure S12.** **(A)** Profiles for 16/23S rRNAs in *E. coli* and digitonin-syn3A lysates were found similar (samples were run on 1% denaturing agarose, MOPS buffer, stained with ethidium bromide). Such resemblance to rRNA profile of *E. coli* lysates was not observed for mycoplasma lysate preparation using other protocols. Here, most native rRNAs were degraded during cell lysis and/or during subsequent processing steps. The control mRNA is about 800 nucleotides long. The upper bands correspond to the 16S and 23S rRNA (about 1,500 and 3,000 nt). RNA was isolated by hot phenol extraction. **(B)** Despite the increase in the concentration of DNA template (T7-eGFP plasmid), no signal of GFP protein was detected for CFE reaction with digitonin-Syn3A lysate. **(C)** Transcription detected for digitonin-Mcap lysate took place at a similar level (few hundreds of fluorescence units for Spi2-DFHBI-1T signal) compared to trypsin-Mcap lysate (**Figure 3B**).

| **(A)** | **(B)** |
| --- | --- |
| 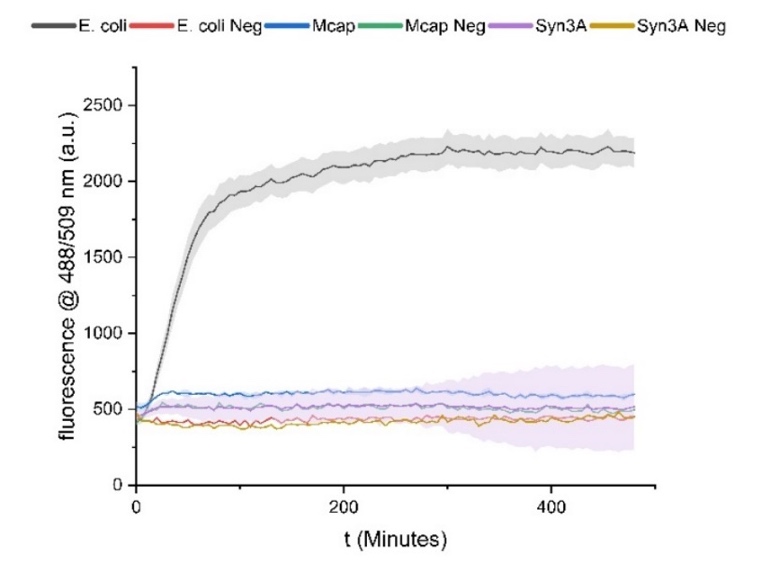 | 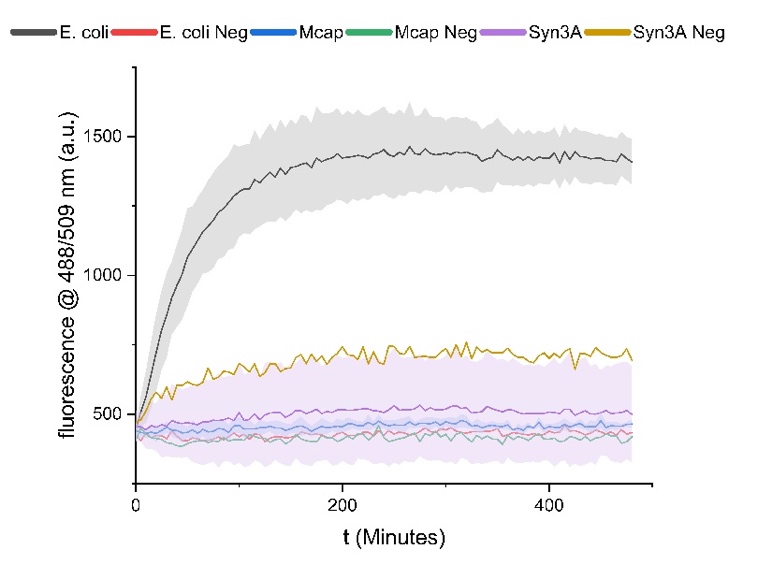 |
| **(C)** |  |
| **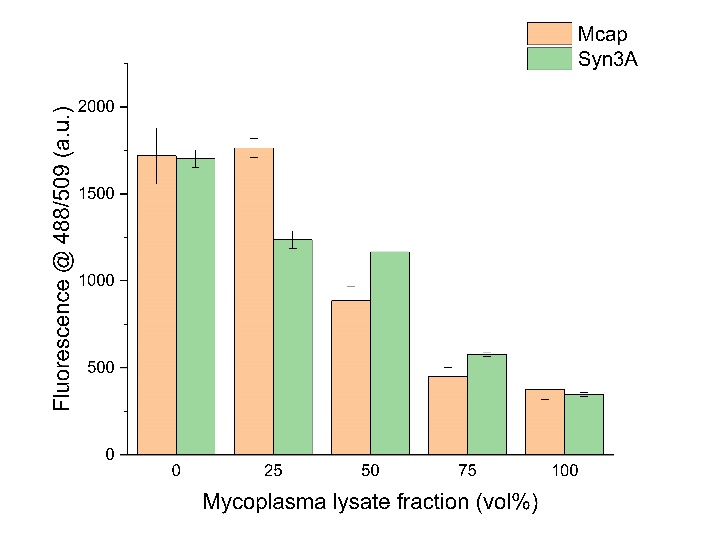** |  |

**Figure S13.** Effect of different RNase inhibitors on eGFP production in mycoplasma CFE. Negative control reactions were supplemented with water instead of DNA template. (**A**) Effect of 60 U/150 µL of Superase-In RNase inhibitor (Thermo Fisher) on eGFP production in Mcap and Syn3A CFE. (**B**) Effect of 60 U/150 µL of RNase Off (Biovision) on eGFP production in Mcap and Syn3A CFE. (**C**) Effect of RNase Off in increasing concentrations of Mcap or Syn3A CFE mixed with functional *E. coli* CFE. (0=100% *E. coli* CFE, 100=100% Mcap or Syn3A CFE)

**Table S1.** List of mycoplasma CFE reaction components with final concentrations. In general, mycoplasma CFE utilized components similar to the *E. coli* CFE reaction mix (2, 3) except for *S. cerevisiae* tRNA. For the preparation of the amino acid stock solutions, each amino acid was dissolved in 5 M KOH and the final pH was adjusted to 6.52 with acetic acid except for arginine hydromonochloride, which was dissolved in water.

| **Component** | **Final concentration in CFE reaction** |
| --- | --- |
| HEPES | 50 mM, pH 8.0 |
| ATP | 1.5 mM, pH 7.5 |
| GTP | 1.5 mM, pH 7.5 |
| CTP | 0.9 mM, pH 7.5 |
| UTP | 0.9 mM, pH 7.5 |
| tRNA | 0.2 mg/mL, *E. coli or S. cerevisiae* |
| CoA | 0.26 mM |
| NAD | 0.33 mM, pH 7.5-8 |
| cAMP | 0.75 mM, pH 8 |
| Folinic acid | 0.07 mM |
| Spermidine | 1 mM |
| 3-PGA | 30 mM, pH 7.5 |
| DTT | 1 mM |
| Alanine | 1.5 mM  per amino acid |
| Arginine hydromonochloride |  |
| Aspartic acid |  |
| Cysteine |  |
| Glutamic acid |  |
| Glycine |  |
| Histidine |  |
| Isoleucine |  |
| Leucine |  |
| Lysine |  |
| Methionine |  |
| Phenylalanine |  |
| Proline |  |
| Serine |  |
| Threonine |  |
| Tryptophan |  |
| Tyrosine |  |
| Valine |  |
| Asparagine |  |
| Glutamine |  |

**Table S2.** List of annotated nucleases in Syn3A and Mcap genomes.

| **Protein name** | **Essentiality** | **Function** | **Gene locus** | | **Protein**  **Length**  **(AA)** | | **Possible**  **Membrane**  **Association**  **(TMHMM)** | | | **Protein Copies per Syn3A cell** | |
| --- | --- | --- | --- | --- | --- | --- | --- | --- | --- | --- | --- |
|  |  |  | **Syn3A** | **Mcap** |  |  |  |  |  |  |  |
| Ribonuclease M5 | quasi-essential | Ribosome biogenesis | JCVISYN3A_0003 | MCAP_0003 | 177/180 (98%) | no | | 65 | | |  |
|  |  |  |  |  |  |  | |  | | |  |
| Magnesium-dependent 5'-3' exonuclease | quasi-essential | DNA metabolism | JCVISYN3A_0097 | MCAP_007 | 258/299 (86%) | | no | | 71 | | |
| Exodeoxyribonuclease VII small subunit | quasi-essential | DNA metabolism | JCVISYN3A_0105 | MCAP_0056 | 66/71 (93%) | | no | | 7 | | |
| Exodeoxyribonuclease VII large subunit | non-essential | DNA metabolism | JCVISYN3A_0106 | MCAP_0057 | 338/468 (72%) | | no | | 15 | | |
| Magnesium-dependent 5'-3' exonuclease | essential | DNA repair | JCVISYN3A_0109 | MCAP_0060 | 278/289 (96%) | | no | | 22 | | |
| NanoRNAse | essential | degradation of oligoribonucleotides | JCVISYN3A_0139 | MCAP_0142 | 361 299/315 (95%) | | no | | 187 | | |
| Putative pre-16S rRNA nuclease | quasi-essential | 16S rRNA maturation | JCVISYN3A_0215 | MCAP0215 | 136/143 (95%) | | no | | 49 | | |
| Excinuclease ABC subunit C | non-essential | DNA repair | JCVISYN3A_0254 | MCAP_0252 | 537/584 (92%) | | no | | 58 | | |
| RNase J family beta-CASP ribonuclease | quasi-essential | RNA turnover | JCVISYN3A_0257 | MCAP_0255 | 561/583 (96%) | | no | | 335 | | |
| Double-stranded RNA binding RNase HI | quasi-essential | DNA replication | JCVISYN3A_0283 | MCAP_0319 | 168/206 (82%) | | no | | 38 | | |
| **Ribonuclease HII*** | essential | RNA metabolism | JCVISYN3A_0296 | MCAP_0332 | 169/214 (79%) | | **yes** | | **30** | | |
| RNase P RNA component class B | essential | tRNA maturation | JCVISYN3A_0356 | MCAP_0553 | 333/340(98%) | | no | |  | | |
| **Ribonuclease Y** | quasi-essential | RNA metabolism | JCVISYN3A_0359 | MCAP_0550 | 464/509 (91%) | | **yes** | | **206** | | |
| Exodeoxyribonuclease 7 large subunit | essential | DNA repair | JCVISYN3A_0373 | MCAP_0536 | 465/535 (87%) | | no | | 48 | | |
| rRNA maturation RNase | quasi-essential | rRNA maturation RNAse | JCVISYN3A_0402 | MCAP_0508 | 151/164 (92%) | | no | | 46 | | |
| Ribonuclease III | essential | RNA metabolism | JCVISYN3A_0418 | MCAP_0492 | 193/232 (83%) | | no | | 59 | | |
| Putative 3'-5' exoribonuclease | essential | RNA metabolism | JCVISYN3A_0437 | MCAP_0473 | 285/326 (87%) | | no | | 517 | | |
| Exonuclease III | quasi-essential | DNA repair | JCVISYN3A_0530 | MCAP_0382 | 166/202 (82%) | | no | | 196 | | |
| Ribonuclease J | essential | RNA metabolism | JCVISYN3A_0600 | MCAP_0623 | 595/611 (97%) | | no | | 120 | | |
| Ribonuclease R | quasi-essential | RNA metabolism | JCVISYN3A_0775 | MCAP_0097 | 688/704 (98% | | no | | 140 | | |
| Excinuclease ABC subunit A | non-essential | DNA repair | JCVISYN3A_0824 | MCAP_0774 | 943/946 (99%) | | no | | 144 | | |
| Excinuclease ABC subunit B | non-essential | DNA repair | JCVISYN3A_0825 | MCAP_0773 | 662/665 (99% | | no | | 49 | | |
| Ribonuclease PH | non-essential | RNA metabolism | JCVISYN3A_0853 | MCAP_0785 | 224/239 (94%) | | no | | 7 | | |
| Ribonuclease P protein component | essential | tRNA maturation | JCVISYN3A_0909 | MCAP_0869 | 99/109 (91%) | | no | | 6 | | |
| Ribonuclease HII* | - | RNA degradation in RNA/DNA hybrids | - | MCAP_0542 | 238 | | no | |  | | |
| 5'-3' exonuclease | - | DNA metabolism | - | MCAP_0047 | 304 | | no | |  | | |
| AlwI family type II restriction endonuclease | - | Restriction endonuclease | - | MCAP_0050 | 525 | | no | |  | | |
| Ribonuclease H | - | RNA degradation in RNA/DNA hybrids | - | MCAP_0319 | 206 | | no | |  | | |
| Bifunctional oligoribonuclease/PAP phosphatase NrnA | - | RNA metabolism | - | MCAP_0142 | 206 | | no | |  | | |
| HNH endonuclease | - | Endonuclease activity | - | MCAP_0725 | 148 | | no | |  | | |
|  |  | **Total nuclease-related genes** | **24** | **30** |  | |  | |  | | |
|  |  | **Unique genes per strain** | 0% | 23% |  | |  | |  | | |
|  |  | **Overlap with JCVI-syn3A** | **-** | 77% |  | |  | |  | | |

*JCVISYN3A_0296 is annotated as an RNase HII based on secondary structure element alignment **(4). The protein, which has four transmembrane helices, does not align with any RNase HII protein based on BLASTP. Mcap and *M. mycoides* (JCVI-syn1.0) both encode proteins that are similar to characterized RNase HII proteins (MCAP_0542 and MMSYN1_0367). RNase HII is not essential and thus not included in Syn3A. Protein copies per JCVI-syn3A cell are from Breuer et al. (5).**

**Table S3.** Available literature (6–9) describing specific conditions for activation and inhibition of nucleases and membrane-associated nucleases for several mycoplasma species.

| **Strain** | **Nuclease type** | **Nuclease activation** | **Nuclease inhibition** |
| --- | --- | --- | --- |
| *M. genitalium* (6) | Endo- / Exo- /  membrane-associated (MG_128) | 10 mM Ca^2+^  10 mM Mg^2+^ | 0.5/20 mM Mn^2+^  0.1-20 mM Zn^2+^ |
| *M. capricolum* (7) | Membrane-associated | 10 mM Mg^2+^ | **2 mM Ca^2+^/Mg^2+^**  **5 mM Ca^2+^/Mg^2+^** |
| *M. hyorhinis* (8) | Endonucleases (similar to apoptotic nucleases in eukaryotes) | 1 mM Ca^2+^/Mg^2+^  5mM Ca^2+^/Mg^2+^  1 mM Ca^2+^/  5 mM Mg^2+^ | 1 mM Ca^2+^/5 mM Mn^2+^  10 mM Zn^2+^ in 1 mM Ca^2+^/Mn2+ |
| *M. penetrans* (9) | Endo- / Membrane-associated (50 kDa) | 2 mM Ca^2+^  5 mM Mg^2+^  pH 7-8 | Mn^2+^, Zn^2+^, heparin, pepstatin |

**Table S4.** List of primers utilized in this work.

| **Primer name** | **Sequence** |
| --- | --- |
| **Cloning and Sanger sequencing** | |
| eGFP_F (Mcap codon usage) | GAAATtaatacgactcactatagg |
| eGFP_R (Mcap codon usage) | cagccggatCTTAAATTCCAGCAGC |
| Spi2_F | GCTAGCTTCCCTCTTGAAATAATTAGAGAGG |
| Spi2_R | CCGCTAGGATCAAGCCTTCGAATTCG |
| mCherry_F (Mcap codon usage) | GAGAAAGGAAATATAACTAGTATGGTATCAAAAGG |
| mCherry_R (Mcap codon usage) | GGTGGTATGGATGAACTATATAAATAATGCATAA |
| **RT-qPCR** | |
| 23S_F | GAAGTAGCGCCATAGAGGGT |
| 23S_R | CTTTCCCTCACGGTACTGGT |

**Table S5.** List of coding sequences utilized in this project.

| **Malachite Green aptamer** |
| --- |
| atggcatctccacctcctcgcggtccgacctgggcatccgaaggaggacgtcgtccactcggatggctaagggagagctcggatccggctgctaacaaagcccgaaaggaagctgagttggctgctgccaccgctgagcaataac |
| **Spinach2 aptamer** |
| GATGTAACTGAATGAAATGGTGAAGGACGGGTCCAGTAGGCTGCTTCGGCAGCCTACTTGTTGAGTAGAGTGTGAGCTCCGTAACTAGTTACATC |
| **Broccoli aptamer** |
| TAATACGACTCACTATAGGGTTGCCATGTGTATGTGGGAGACGGTCGGGTCCAGATATTCGTATCTGTCGAGTAGAGTGTGGGCTCCCACATACTCTGATGATCCTTCGGGATCATTCATGGCAA |
| **eGFP (codon-optimized for Mcap) (CG% 26.7)** |
| ATGGAATTATTTACTGGAGTTGTTCCAATTTTAGTTGAATTAGATGGAGATGTTAATGGACATAAATTTTCAGTTTCAGGAGAAGGAGAAGGAGATGCTACTTATGGAAAATTAACTTTAAAATTTATTTGTACTACTGGAAAATTACCAGTTCCATGGCCAACTTTAGTTACTACTTTAACTTATGGAGTTCAATGTTTTTCAAGATATCCAGATCATATGAAACAACATGATTTTTTTAAATCAGCTATGCCAGAAGGATATGTTCAAGAAAGAACTATTTTTTTTAAAGATGATGGAAATTATAAAACTAGAGCTGAAGTTAAATTTGAAGGAGATACTTTAGTTAATAGAATTGAATTAAAAGGAATTGATTTTAAAGAAGATGGAAATATTTTAGGACATAAATTAGAATATAATTATAATTCACATAATGTTTATATTATGGCTGATAAACAAAAAAATGGAATTAAAGTTAATTTTAAAATTAGACATAATATTGAAGATGGATCAGTTCAATTAGCTGATCATTATCAACAAAATACTCCAATTGGAGATGGACCAGTTTTATTACCAGATAATCATTATTTATCAACTCAATCAGCTTTATCAAAAGATCCAAATGAAAAAAGAGATCATATGGTTTTATTAGAATTTGTTACTGCTGCTGGAATTTGA |
| **eGFP (*E. coli* sequence) (GC% 61.1)** |
| ATGGAGCTTTTCACTGGCGTTGTTCCCATCCTGGTCGAGCTGGACGGCGACGTAAACGGCCACAAGTTCAGCGTGTCCGGCGAGGGCGAGGGCGATGCCACCTACGGCAAGCTGACCCTGAAGTTCATCTGCACCACCGGCAAGCTGCCCGTGCCCTGGCCCACCCTCGTGACCACCCTGACCTACGGCGTGCAGTGCTTCAGCCGCTACCCCGACCACATGAAGCAGCACGACTTCTTCAAGTCCGCCATGCCCGAAGGCTACGTCCAGGAGCGCACCATCTTCTTCAAGGACGACGGCAACTACAAGACCCGCGCCGAGGTGAAGTTCGAGGGCGACACCCTGGTGAACCGCATCGAGCTGAAGGGCATCGACTTCAAGGAGGACGGCAACATCCTGGGGCACAAGCTGGAGTACAACTACAACAGCCACAACGTCTATATCATGGCCGACAAGCAGAAGAACGGCATCAAGGTGAACTTCAAGATCCGCCACAACATCGAGGACGGCAGCGTGCAGCTCGCCGACCACTACCAGCAGAACACCCCCATCGGCGACGGCCCCGTGCTGCTGCCCGACAACCACTACCTGAGCACCCAGTCCGCCCTGAGCAAAGACCCCAACGAGAAGCGCGATCACATGGTCCTGCTGGAGTTCGTGACCGCCGCCGGGATCTAA |

**References**

1. Mariscal,A.M., Kakizawa,S., Hsu,J.Y., Tanaka,K., González-González,L., Broto,A., Querol,E., Lluch-Senar,M., Piñero-Lambea,C., Sun,L., *et al.* (2018) Tuning Gene Activity by Inducible and Targeted Regulation of Gene Expression in Minimal Bacterial Cells. *ACS Synth. Biol.*, **7**, 1538–1552.

2. Garamella,J., Marshall,R., Rustad,M. and Noireaux,V. (2016) The All E. coli TX-TL Toolbox 2.0: A Platform for Cell-Free Synthetic Biology. *ACS Synth. Biol.*, **5**, 344–355.

3. Sun,Z.Z., Hayes,C.A., Shin,J., Caschera,F., Murray,R.M. and Noireaux,V. (2013) Protocols for implementing an Escherichia coli based TX-TL cell-free expression system for synthetic biology. *J. Vis. Exp.*, 10.3791/50762.

4. Yang,Z. and Tsui,S.K.W. (2018) Functional Annotation of Proteins Encoded by the Minimal Bacterial Genome Based on Secondary Structure Element Alignment. *J. Proteome Res.*, **17**, 2511–2520.

5. Breuer,M., Earnest,T.M., Merryman,C., Wise,K.S., Sun,L., Lynott,M.R., Hutchison,C.A., Smith,H.O., Lapek,J.D., Gonzalez,D.J., *et al.* (2019) Essential metabolism for a minimal cell. *Elife*, **8**.

6. Li,L., Krishnan,M., Baseman,J.B. and Kannan,T.R. (2010) Molecular cloning, expression, and characterization of a Ca^2+^-dependent, membrane-associated nuclease of Mycoplasma genitalium. *J. Bacteriol.*, **192**, 4876–84.

7. Minion,F.C., Jarvill-Taylor,K.J., Billings,D.E. and Tigges,E. (1993) Membrane-associated nuclease activities in mycoplasmas. *J. Bacteriol.*, **175**, 7842–7.

8. Paddenberg,R., Weber,A., Wulf,S. and Mannherz,H.G. (1998) Mycoplasma nucleases able to induce internucleosomal DNA degradation in cultured cells possess many characteristics of eukaryotic apoptotic nucleases. *Cell Death Differ.*, **5**, 517–28.

9. Bendjennat,M., Blanchard,A., Loutfi,M., Montagnier,L. and Bahraoui,E. (1997) Purification and characterization of Mycoplasma penetrans Ca2+/Mg2+- dependent endonuclease. *J. Bacteriol.*, **179**, 2210–2220.
