# Supplementary material for: Traditional protocols and optimization methods lead to absent expression in a mycoplasma cell-free gene expression platform
Source: Synth Biol (Oxf). 2022 May 21;7(1):ysac008. doi: 10.1093/synbio/ysac008 (PMC9239315; doi:10.1093/synbio/ysac008)
Supplement: ysac008_Supp [file ysac008_supp.zip › JCVI-CodexDNA MTA for minimal cell.template.docx]

**MATERIAL TRANSFER AGREEMENT**

This Agreement dated and effective as of the date of the last signature on this agreement is by and between CODEX, J. Craig Venter Institute (“Provider”) and and [___________________] (“Recipient”).

CODEX owns certain materials, described as follows: Bacterial strains *Mycoplasma mycoides* subspecies c*apri* strain GM12 (also called JCVI-syn1.0), JCVI-syn3.0, JCVI-syn3A, JCVI-syn3B and various derivatives of those organisms that have been genetically modified at the JCVI, and any information provided to Recipient by SGIDNA or Provider, as more specifically described in Exhibit A (“Material”). Recipient is interested in using the Material and related data solely for the purpose of __________________________________________________. (“Recipient Purpose”).

Acknowledging the adequacy of the consideration exchanged, CODEX and Recipient agree to the following terms.

1. CODEX agrees to transfer a sample of Material to Recipient. Material shall be transferred on behalf of CODEX by Provider to Recipient and Recipient agrees to receive and only use the Material subject to and in accordance with the obligations set forth in this Agreement.
2. Recipient agrees that the Material: (a) will be used in compliance with all applicable statutes and regulations, such as, without limitation, those relating to research involving the use of animals or recombinant DNA, to use and handling of specific bacterial strains pursuant to USDA requirements, and to U.S. export control laws; (b) will not be used in human subjects, in clinical trials, or for diagnostic purposes involving human subjects without the written consent of CODEX; (c) will be used only in Recipient’s laboratory; and (d) will not be transferred or otherwise made available to anyone outside of Recipient’s laboratory without the express prior written consent of CODEX.
3. If the Material requires the Recipient to have USDA authorization to work with the Material, *e.g*., Material consisting of, derived from or classified as *Mycoplasma* strains, Recipient shall provide verification of such authorization to CODEX prior to transfer of Material.
4. CODEX will retain ownership of (a) the Material, all expression products thereof, and all parts, progeny, unmodified derivatives and any CODEX uses of any of the foregoing, (b) any Material contained or incorporated in modifications created by Recipient, and (c)  all intellectual property rights in and to each of the foregoing (collectively, “CODEX’s Rights”). Recipient shall take such actions as may be reasonably necessary to vest, secure, perfect, protect or enforce the right, title and interest of CODEX in and to CODEX’s Rights, at CODEX’s cost. Without limiting the generality of the foregoing, CODEX shall have the right to exploit, license and otherwise transfer CODEX’s Rights.

Recipient acknowledges that the Material is or may be the subject of a patent application. Except as provided in this Agreement, no express or implied licenses or other rights are provided to the Recipient under any patents, patent applications, trade secrets or other proprietary rights of CODEX, including any altered forms of the Material made by CODEX. In particular, no express or implied licenses or other rights are provided to use the Material, modifications, or any related patents of CODEX for Commercial Purposes.

Recipient will promptly notify CODEX of any results, innovations, inventions or discoveries that are conceived and reduced to practice in the performance of the Recipient Purpose, whether patentable or not (“Inventions”).  Except as provided herein, Recipient retains all right, title, and ownership in and to Inventions. Recipient hereby grants CODEX a perpetual, worldwide, royalty-free, non-transferrable, non-sublicenseable, non-exclusive license to practice Inventions for CODEX’s internal research purposes. CODEX; and (ii) the first right of negotiation for an exclusive, worldwide, royalty-bearing license (with the right to grant sublicenses through multiple tiers) under any such Inventions (together with all patent and other intellectual property rights therein and thereto).

Recipient will provide (i) written notice to CODEX prior to the submission of any patent application covering any Invention, along with copies of all draft patent applications and correspondence to and from the patent office; and (ii) good faith consideration to any comments or suggestions received from CODEX regarding such patent applications and correspondence.

1. Subject]to the terms and conditions of this Agreement, Recipient shall have the right to use data, information, discoveries, inventions and other results directly or indirectly derived from or relating to the use of the Material by Recipient for the Recipient Purpose, for its own internal, noncommercial, research purposes.

Recipient will use Material only for the Recipient Purpose and not for any other purpose. Commercial Purposes include the sale, lease, license, or other transfer of the Material or any part, progeny, modification or derivative thereof. Commercial Purposes shall also include uses of the Material or any part, progeny, modification or derivative thereof by any organization, including Recipient, to perform contract research or perform a service for a fee, to screen compound libraries, to produce or manufacture products for general sale, or to conduct research activities that result in any sale, lease, license, or transfer of Material or any part, progeny, modification or derivative thereof. Recipient will not reverse engineer any of the Material.

1. Recipient will acknowledge CODEX as the source of Material in publications or other presentations arising from Recipient’s use of Material. Recipient shall provide a copy of proposed publication or presentation to CODEX for a review period not to exceed thirty (30) days prior to publication.CODEX Furthermore, the Recipient agrees to provide a report of the results generated from the use of the Material within thirty (30) days after completion of such Research Purpose or upon termination or expiration of this Agreement.

Should either party wish to publish or present work or results undertaken or obtained in connection with Recipient Purpose, such party will furnish the other parties with a copy of the manuscript, abstract or presentation disclosing such work or results prior to submission thereof not less than thirty days prior to publication. If Confidential Information (as defined in Section 12)] of another party is identified in the proposed publication during such thirty (30) day period, consideration of removal of such Confidential Information shall be made in good faith. If the non-publishing parties have not responded within thirty (30) days after the publishing party provides the copy of the manuscript, abstract or presentation, then the publishing party may proceed with publication or disclosure without further delay. If patenable information is disclosed in the proposed publication, the submitting party will delay publication to allow a reasonable period of time to file a patent application; however, in no case shall a delay period last longer than ninety (90) days from submission to the other party.  Each party shall acknowledge the other party(ies) in any publication in accordance with scientific custom, unless the other parties otherwise instructs. Notwithstanding the above, CODEX shall not be required to request permission for publication or disclosure from Provider.

1. Recipient acknowledges that the Material is experimental and is provided to Recipient WITHOUT ANY WARRANTIES, EXPRESS OR IMPLIED, INCLUDING ANY WARRANTY OF TITLE, MERCHANTABILITY, FITNESS FOR A PARTICULAR PURPOSE OR NONINFRINGEMENT. Except as explicitly provided by this Agreement, no license or other rights (express or implied) are granted by CODEX. The parties acknowledge that any results provided by Recipient from Recipient’s performance under this Agreement are shared WITHOUT ANY WARRANTIES, EXPRESS OR IMPLIED, INCLUDING ANY WARRANTY OF TITLE, MERCHANTABILITY, FITNESS FOR A PARTICULAR PURPOSE OR NONINFRINGEMENT.
2. Indemnification.
   1. Recipient shall indemnify, defend, and hold harmless CODEX and its respective officers, employees, contractors, and agents (the “CODEX Indemnitees”) from and against any and all third party liability, damage, loss, cost or expense (including attorney's fees), of whatsoever kind or nature, which the CODEX Indemnitees may hereafter incur, or be required to pay, as a result of Recipient's handling or storage of the Materials; provided that Recipient’s obligations pursuant to this Section 13(a) shall not apply to the extent such claims or suits result from the gross negligence or willful misconduct of any of CODEX Indemnitees as determined by a court of law.
   2. CODEX shall indemnify, defend and hold harmless Recipient and its respective trustees, officers, faculty, students, employees, contractors and agents (the “Recipient Indemnitees”) from and against any and all third party liability, damage, loss, cost or expense (including reasonable attorneys’ fees), which the Recipient Indemnitees may hereafter incur, or be required to pay as a result of CODEX’s use of Research Results or Inventions, provided that CODEX obligations pursuant to this Section 13(b) shall not apply to the extent such claims or suits result from the gross negligence or willful misconduct of any of Recipient Indemnitees as determined by a court of law.
   3. As a condition to an Indemnitee’s right to receive indemnification under this Section 13, the Indemnitee shall: (a) promptly notify the indemnifying party (“Indemnifying Party”) when it becomes aware of a claim or suit for which indemnification may be sought pursuant hereto; (b) cooperate with the Indemnifying Party in the defense, settlement or compromise of such claim or suit; and (c) permit the Indemnifying Party to control the defense, settlement or compromise of such claim or suit, including the right to select defense counsel. In no event, however, may the Indemnifying Party compromise or settle any claim or suit in a manner which (a) admits fault or negligence on the part of the Indemnitee; or (b) commits the Indemnitee to take, or forbear to take, any action, without the prior written consent of the Indemnitee. The Indemnitee shall reasonably cooperate with the Indemnifying Party and its counsel in the course of the defense of any such suit, claim or demand.

CODEXCODEXCODEX.

1. Recipient agrees not to use CODEX’s name without CODEX’s prior written consent CODEX agrees not to use Recipient’s name, or the name of any trustee, officer, faculty member, student or employee thereof, including Principal Investigator, without Recipient’s prior written consent.
2. This Agreement represents the entire understanding between the parties with respect to its subject matter and may only be changed in a writing signed by both parties. This Agreement is not assignable by Recipient; CODEX may freely assign this Agreement and any of its rights and obligations hereunder. This Agreement shall be governed by and construed in accordance with the laws of the State of California without recourse to its conflicts of laws provisions. Each individual signing for a corporate entity hereby personally warrants his or her legal authority to bind that entity.
3. The term of this Agreement is for a period of three (3) years. This Agreement may be terminated by either party by providing ten (10) days notice to the party in writing. Upon the completion of the Recipient Purpose, expiration or termination of this Agreement, Recipient shall destroy or return the Material as requested by CODEX. If Recipient breaches this Agreement and does not cure such breach within ten (10) days of CODEX’s written notice, then Recipient agrees to immediately cease any use of the Material and all related information, at which time all of the Materials and any modifications, extracts and derivatives and related information provided by CODEX or developed by Recipient or on Recipient’s behalf will be provided to CODEX or entirely destroyed at CODEX’s request. The provisions of Sections 4, 6, 7, 8, 9, 10 and 11 shall survive any expiration or termination of this Agreement.
4. All information disclosed by one party (“Disclosing Party”) to the other party (“Receiving Party”) clearly marked as confidential by Disclosing Party, or if not clearly marked, identified at the time of the disclosure as confidential by the Disclosing Party, (and in the case of CODEX, including without limitation any extracts supplied by CODEX which is related to Material that is supplied by CODEX) (“Confidential Information”), and any of Disclosing Party’s Confidential Information that is incorporated in information of the Receiving Party shall be deemed to belong to Disclosing Party, CODEX and to have been disclosed or provided to Receiving Party in confidence. All parties agree agrees to preserve the confidential status of the other parties’ confidential information, and shall not be used or further disclosed to any third party for any purpose other than the Recipient Purpose, or otherwise expressly permitted herein. The obligations of both Parties to maintain confidentiality under this Agreement will survive its expiration or termination and will endure for five (5) years from date of disclosure. These confidentiality obligations do not apply to any information that:
   1. was known to Receiving Party prior to the receipt from Disclosing Party or that is developed by Receiving Party independently of the Disclosing Party’s confidential information, as shown by competent evidence;
   2. becomes known to the public not as a result of any action or inaction by Receiving Party;
   3. the Receiving Party acquires from a third party who has the right to disclose such information to Receiving Party without restriction; and
   4. the Receiving Party is required to disclose information by law, order or regulation of a governmental agency or a court of competent jurisdiction or international authority and has given Disclosing Party as much advance notice of the disclosure as is reasonably practicable, and reasonably cooperated with the Disclosing Party’s lawful efforts to suppress or limit such disclosure.

**[Recipient]**

_______________________________________ Date: ___________________________

Name: Title:

**CODEX**

_______________________________________ Date: ___________________________

Name:

Title of Authorized Signatory:

**J. Craig Venter Institute**

_______________________________________ Date: ___________________________

Name: Richard Scheuermann, Ph. D.

Title: Director, La Jolla Campus

READ AND UNDERSTOOD BY:

_______________________________________ Date: ___________________________

Recipient Scientist

Exhibit A

**Description of Material:**

Bacterial strains JCVI-syn1.0, JCVI-syn3.0, JCVI-syn3A, JCVI-syn3B, and derivatives of those strains made by the JCVI. Yeast strains containing the genomes of JCVI-syn1.0, JCVI-syn3.0, JCVI-syn3A, JCVI-syn3B and derivateves of those strains made by the JCVI. Plasmids used for manipulation of the above strains of bacteria and yeast. Mycoplasma capricolum subspecies capricolum strain California kid mutated to disrupt its restriction endonuclease (this is the recipient cell in genome transplantation reactions).
